# A new technique for stain-marking of seeds with safranine to track seed dispersal and seed bank dynamics

**DOI:** 10.3389/fpls.2022.959046

**Published:** 2022-08-08

**Authors:** Zheng Zhang, Xinglei Shi, Ruhai Li, Sheng Qiang

**Affiliations:** ^1^Weed Research Laboratory, Nanjing Agricultural University, Nanjing, China; ^2^Institute of Plant Protection and Soil Fertilizer, Hubei Academy of Agricultural Sciences, Wuhan, China

**Keywords:** stain method, *ex situ* and *in situ* staining, staining intensity, HSV color space, seed marking and tracking, seed dispersal, seed identification

## Abstract

Accurate tracking of seed dispersal is critical for understanding gene flow and seed bank dynamics, and for predicting population distributions and spread. Available seed-tracking techniques are limited due to environmental and safety issues or requirements for expensive and specialized equipment. Furthermore, few techniques can be applied to studies of water-dispersed seeds. Here we introduce a new seed-tracking method using safranine to stain seeds/fruits by immersing in (*ex situ*) or spraying with (*in situ*) staining solution. The hue difference value between pre- and post-stained seeds/fruits was compared using the HSV color model to assess the effect of staining. A total of 181 kinds of seeds/fruits out of 233 tested species of farmland weeds, invasive alien herbaceous plants and trees could be effectively stained magenta to red in hue (320–360°) from generally yellowish appearance (30–70°), in which the other 39 ineffectively-stained species were distinguishable by the naked eye from pre-stained seeds. The most effectively stained seeds/fruits were those with fluffy pericarps, episperm, or appendages. Safranine staining was not found to affect seed weight or germination ability regardless of whether seeds were stained *ex situ* or *in situ*. For 44 of 48 buried species, the magenta color of stained seeds clearly remained recognizable for more than 5 months after seeds were buried in soil. Tracking experiments using four species (*Beckmannia syzigachne*, *Oryza sativa* f. spontanea, *Solidago Canadensis*, and *Acer buergerianum*), representing two noxious agricultural weeds, an alien invasive plant, and a tree, respectively, showed that the safranine staining technique can be widely applied for studying plant seed dispersal. Identifying and counting the stained seeds/fruits can be executed by specially complied Python-based program, based on OpenCV library for image processing and Numpy for data handling. From the above results, we conclude that staining with safranine is a cheap, reliable, easily recognized, automatically counted, persistent, environmentally safe, and user–friendly tracking-seed method. This technique may be widely applied to staining most of the seed plant species and the study of seed dispersal in arable land and in disturbed and natural terrestrial or hydrophytic ecological systems.

## Introduction

Studies of seed and fruit dispersal are one of the most important aspects of plant population ecology. For both invasion and native plants, seed dispersal influences population dynamics, (re)establishment, species diversity, community structure, genetic structure, and species interactions (Bohrer et al., 2005; García et al., 2007; Beckman et al., 2012; Emmerson et al., 2012). To understand plant dispersal, a detailed knowledge of the processes that determine it is critical. However, it is technically difficult to determine the distribution of dispersed seeds and to directly relate seed dispersal to the plant community. The primary obstacle is how to assign seed already deposited in the soil a specific mother plant. In rare cases, direct observation of the dispersal process is possible, but in most situations, linking dispersed seeds with parent plants requires some method of tagging seeds before they leave the source plants (Nathan and Muller-Landau, 2000; Wang and Smith, 2002; Nathan, 2006). Tracking seed dispersal from specific plant sources at longer distances is even more difficult. Thus, any technique that allows researchers to easily identify dispersed seeds from specific source plants would help to better understand dispersal biology (Will and Tackenberg, 2008).

Several methods have been developed for the study of seed dispersal; these include the surgical implantation of magnets (Alverson and Díaz, 1989), attachment of colored threads or tags to seeds (Forget, 1992; Hirsch et al., 2012a; Deng et al., 2020), radioisotope labeling (Vander Wall, 1994), fluorescent microsphere and dye powder labeling (Levey and Sargent, 2000), stable isotope (^15^N) analysis (Carlo et al., 2009; Wang et al., 2021), attachment passive integrated transponders (PITs) tags (Suselbeek et al., 2013); and molecular or genetic marking (Ouborg et al., 1999; Godoy and Jordano, 2001; Grivet et al., 2005; Hardesty et al., 2006). These methods can help researchers to match dispersed seeds and seedlings with their parent plants in some situations; however, most are prohibitively expensive, logistically intractable, or excessively laborious and time consuming.

Here we introduce a new method to track seed dispersal based on seed staining by spraying with or soaking in safranine solution, which is usually used as a stain for textiles and as a stain for various scientific procedures, such as in histology and cytology. This new method will help tackle one of the methodological bottlenecks in the study of seed dispersal biology, as it shows the high applicability in various dispersal modes and it is cost effective and environmentally friendly compared with other marking techniques.

After the retrieving of stained seeds, the rapid identification and count of the stained seeds is a key step for the application of the staining technique. Here we introduced an identification and count method based on the OpenCV^1^ which is an open source computational infrastructure library that provides a rich set of image-analysis algorithms in the domain of computer vision. OpenCV offers, for example, feature-extraction algorithms that can identify notable structures from images, feature matching and tracking algorithms (Eliceiri et al., 2012). The OpenCV based programs can be used in many applications, such as clinical medicine and disease diagnosis (Gao et al., 2015; Yenilmez et al., 2016), network extraction from images (Dirnberger et al., 2015), the biological research of cell (Rosendahl et al., 2018). But the application of OpenCV-based program in plant ecology is limited.

## Materials and methods

### Staining *ex situ*

Three categories of diaspores (fruits or seeds), totally 233 species, were selected from native and introduced plant species present in China: main and important native farmland weeds (135 species, seeds of 43 species and fruits of 92 species) (Supplementary Table 1), serious infested invasive alien herbaceous plants (63 species, seeds of 17 species and fruits of 46 species) (Supplementary Table 2), and important and rare native woody plants (35 species, seeds of 12 species and fruits of 23 species) (Supplementary Table 3). Mature diaspores of the 233 species were collected and air dried at room temperature for 15–20 days in the laboratory.

A subset of 13 representative plant species was used to compare staining concentration, staining time (operation time), and drying time with two staining approaches: *in situ* (spraying) or *ex situ* (immersion). To determine the suitable dilution for seed/fruit staining, a preliminary experiment was conducted where safranine (Sinopharm Chemical Reagent Co., Ltd., China) solutions were prepared at 0.5, 1.0, and 2.0% (w/v) in 50% ethanol. Diaspores of the 13 selected species were soaked separately in safranine solution at the three concentrations at varying times, according to their texture and size, for up to an hour, and then air dried. Herbaceous and ligneous diaspores were soaked for 10–15 and 60 min, respectively; others were soaked for 30 min. When soaking, the diaspores were stirred to fully expose them to the staining solution. Digital photographs of the stained and unstained diaspores were taken under the same illumination and exposure time to evaluate the staining effect. Based on this preliminary experiment, the concentration of safranine staining solution and the soaking time were established for subsequent use on the remaining 220 plant species. Digital photos were taken for all stained and unstained diaspores.

### Staining *in situ*

The same 13 species as were used in the *ex situ* staining study were also used for tests of *in situ* staining. At plant maturity, the diaspores still attached to their mother plants were sprayed with safranine with a hand-operated mister to complete uniform wetness. The diaspores were sprayed on sunny days and allowed to dry naturally for about 15 min before being photographed as before.

### Influence of staining on seed weight and germination

The diaspores of the 13 species used in both *in situ* and *ex situ* staining experiments were tested to evaluate the influence of staining on diaspores weight and germination. To determine the 1,000-diaspore weight, except for *Q. acutissima*, the unstained, *in situ* stained and *ex situ* stained diaspores were weighed in five replicates, respectively, of 1,000 diaspores per replicate. For *Q. acutissima*, the weight was determined for five batches of 100 acorns per replicate. To determine the germination rate, 100 seeds (20 acorns for *Q. acutissima*) with five replicates per species were placed in 15-cm diameter petri dishes with filter paper moistened by sterilized water and placed in an incubator at appropriate temperatures according to literature (30°C for *C. difformis*, 25°C for *E. crus-galli*, *P. communis*, and *P. stenoptera*, 20°C for *E. ulmoides*, *O. sativa*, *S. canadensis*, *A. buergerianum*, *A. subulatus*, *Q. acutissima*, and *U. parvifolia*, 15°C for *A. japonicas*, 10°C for *B. syzigachne*) with a 12-h photoperiod. Germination was recorded every 2 days until no additional germination occurred.

### Persistence of color on buried seeds

Forty-eight species were selected to assess the durability of seed-staining if they were buried underground. Stained and unstained diaspores, enclosed in separate 0.25 mm mesh nylon bags, were buried 10 cm under the soil surface in a lowland rice field (Jiangsu Province, China) and marked for later retrieval. When buried, diaspore was scattered as homogenously as possible to maximize contact with the soil. At 30, 90, and 150 days after burial, bags were retrieved and at least 50 seeds were collected for evaluation before the bags were returned to the same position in the soil and reburied. Collected seeds were rinsed to remove debris and oven-dried as required at 30 ± 2°C. Digital photos of the dried seeds were taken as described above to evaluate the color change.

### Seed tracking experiments

Four different seed tracking experiments were carried out with *A. buergerianum*, *B. syzigachne*, *O. sativa*, and *S. canadensis* stained using either the *in situ* or *ex situ* technique.

#### Wind dispersal

Wind seed dispersal studies were conducted with *S. canadensis* and *A. buergerianum* in a wasteland in Shanghai, China in November 2014 and in Nanjing, China in October 2017, respectively. Seeds of both species were stained *in situ* as described above prior to shattering. Seed traps were placed along 10 radial axes from the focal plants (with 22.5° angles along the prevailing wind direction and 45° angle at other direction, one plant for *A. buergerianum*, 4-m diameter circular covering area for *S. canadensis* with the density of about 10 plants/m^2^). Tung oil was brushed on the ground every 10 m (0–100 m)/50 m (100–500 m)/100 m (500–2,000 m) along the radial axes to trap *S. canadensis* seeds. Nylon tuck nets set up at ground level every 2 m (0–30 m) along the radial axes were used to trap seeds of *A. buergerianum*.

#### Water dispersal

Several simulation experiments were conducted in June 2010 in Baihu Farm, Anhui Province, China, using seeds stained by the *ex situ* staining method to study the water dispersal dynamics of *B. syzigachne* in an irrigation canal and within a rice field. Stained seeds were released and then tracked during irrigation by systematic sampling at fixed time intervals, and the detailed methods were described by Zhang et al. (2019).

#### Machinery dispersal

A tracking experiment was conducted using seeds stained by the *in situ* staining method to assess dispersal of weedy rice seeds (*O. sativa*) within fields by combine harvester; marked seeds were tracked after harvesting operations along harvesting trails in November 2013 and November 2014 in Suqian and Jintan cities, Jiangsu Province, China, and the detailed methods were described by Gao et al. (2018).

### Assessment of stained vs. unstained seed distinguishability

A set of 136 species (represented by three photographs for each species: stained diaspores only, unstained diaspores only, stained and unstained diaspores mixed together) was selected to visually compare stained and unstained diaspores. All the photographs were divided into 3 groups for the staining status of diaspores. Batch processing of photos was performed by OpenCV, Numpy, and Scikit Image in Python environment for the identification and count of the diaspores. For each photograph, the main treatment steps were (1) color space conversion (GBR to HSV), (2) definition of target color of stained diaspores, (3) mask (binary image) obtaining, (4) morphological process: opening and closing operation, (5) contours discovery and numbering. (Code example is attached as Supplementary Table 1, and the examples of the indemnification of stained diaspores are attached as Supplementary Figure 9.)

### Data analysis

In order to describe the color of plant diaspores (seeds/fruits) before and after staining quantitatively, the HSV (Hue, Saturation, Value) color model (Smith, 1978) was used in this study which is more natural for the human visual system to describe a color image than by the RGB model (Chen et al., 2007).

From each photograph, three randomly selected diaspores were used for individual assessment of its HSV values. One-way analysis of variance (ANOVA) was used to assess the differences between post-stained (*in situ* or *ex situ*) and pre-stained diaspores in the hue of HSV color model, 1,000-seed weight and germination rate. Differences obtained at a level of *p* ≤ 0.05 were considered significant.

The diaspore types, the appendages, and the texture of each species were assigned according to Table 1, and then the Pearson correlation coefficient between the seed types, the appendages, texture, and the HSV of seeds/fruits was calculated.

**TABLE 1:** Assignment of numerical values to the traits of diaspores.

Influence factor	Assignment
Seed or fruit	Seed = 0, fruit = 1
Seed/fruit types	caryopsis = 1, achene or aggregate achenes = 2, nut or nutlet = 3, capsule = 4, legume = 5, silique = 6, cremocarp = 7, utricle = 8, follicle or aggregate follicles = 9, samara = 10, berry = 11, drupe = 12, other = 13
Texture	From soft to hard: membranous = 1, chartaceous = 2, herbaceous = 3, coriaceous = 4, cartilaginous = 5, indurate = 6, others = 7
Appendage	Attached with appendages = 1, absent = 0

The hierarchical clustering (Euclidean distance, complete linkage) based on the HSV of pre- and post-stained diaspores was conducted to classify the color of tested diaspores using R v3.5.1^2^ with flexclust package, and the trees were visualized with FigTree v1.4.3.^3^

The count correct rates of stained diaspores in stained only group and mixed group, and the count correct rates of unstained diaspores in unstained group were calculated to evaluate the efficacy of quick counting method based on OpenCV and Numpy.

The ANOVA and correlation analysis were performed with SPSS 19.0 (IBM Corporation, Armonk, NY, United States), and the figures were drawn using OriginPro 9.0 (Origin Lab Corporation, Northampton, United States).

## Results

### Comparison of two implementation options of *in situ* and *ex situ* staining

These tested plant diaspores varying in size and type were stained magenta to red in hue (320–360°) (Table 2). In general, the hue of post-stained diaspores of the 13 tested species was similar when stained with 1 and 2% safranine solutions. But when the staining solution was 0.5%, the hue of post-stained diaspores of *S. canadensis*, *O. sativa*, *Q. acutissima*, *P. stenoptera*, and *U. parvifolia* (i.e., 5 out of the 13 tested species) was significantly lighter (hue from 0 to 30°) than the hue stained at higher concentrations (from 320 to 360°). Among them, the diaspores of *Q. acutissima* had a hard texture; *O. sativa* had the relative deeper color; *U. parvifolia* had the largest size and original deeper color. Because there was no significant difference in the efficiency in staining by 2.0 and 1.0% safranine solution, the 1.0% safranine solution was the final recommendation. There was no significant difference of staining effect between *ex situ* and *in situ* staining approaches for most species tested, except for *Q. acutissima*. The staining and drying times for *ex situ* staining were much longer than for *in situ* staining. The operation (immersion) time was more than 15 min when stained *ex situ*, with the drying of the soaked diaspores lasting more than 1 day at room temperature. However, for *in situ* staining, the total staining and sun drying time was less than 30 min (15 min for most species).

**TABLE 2:** Comparison of seed colors, staining and drying time.

Species	Fruit type	Main dispersal agents	Hue (°)					Staining/Drying time		Quantity of stained seeds/fruits (grains)	Dispersal distance (m)
			
			Pre-stained	Stained *ex situ*			Stained *in situ*	*In situ*	*Ex situ*		
				
				0.5%	1.0%	2.0%					
*Oryza sativa*	Caryopsis	Machinery	36.93 ± 0.50 b	9.70 ± 3.00 c	345.96 ± 3.05 a	348.37 ± 5.21 a	345.72 ± 3.14 a	15 min	30 min/1 d	>15, 000	>3,000
*Beckmannia syzigachne*	Caryopsis	Water	45.42 ± 0.73 c	357.24 ± 1.73 a	349.53 ± 1.35 a	357.81 ± 1.39 a	345.37 ± 3.92 a	15 min	15 min/1 d	>1, 200, 000	>2,000
*Alopecurus japonicus*	Caryopsis	Water	50.62 ± 11.13 b	335.72 ± 0.67 a	339.86 ± 10.50 a	342.02 ± 2.34 a	331.35 ± 2.44 a	15 min	15 min/1 d	/	/
*Echinochloa crus-galli*	Caryopsis	Water	41.18 ± 3.85 b	307.67 ± 8.61 a	339.12 ± 12.02 a	328.61 ± 7.74 a	355.31 ± 2.81 a	15 min	15 min/1 d	/	/
*Phragmites communis*	Caryopsis	Wind/Water	39.87 ± 7.44 b	353.54 ± 3.05 a	342.66 ± 13.35 a	352.67 ± 0.24 a	334.36 ± 11.02 a	15 min	15 min/1 d	/	/
*Cyperus difformis*	Nutlet	Water	32.51 ± 5.38 b	346.33 ± 0.31 a	354.04 ± 4.58 a	351.76 ± 3.31 a	351.21 ± 2.20 a	15 min	15 min/1 d	/	/
*Solidago canadensis*	Achene	Wind	30.74 ± 3.47 b	2.88 ± 0.83 c	347.24 ± 3.45 a	334.66 ± 7.93 a	342.66 ± 2.03 a	15 min	10 min/1 d	>100, 000, 000	>2,000
*Aster subulatus*	Achene	Wind	31.36 ± 1.93 b	342.62 ± 7.40 a	345.89 ± 7.36 a	347.43 ± 0.23 a	347.29 ± 0.59 a	15 min	10 min/1 d	>5, 000	>20
*Pterocarya stenoptera*	Samara	Wind	27.88 ± 1.53 b	25.56 ± 12.74 b	337.45 ± 5.03 a	334.41 ± 4.58 a	346.04 ± 7.06 a	15 min	30 min/1 d	/	/
*Quercus acutissima*	Nut	Animal	29.86 ± 0.93 b	14.88 ± 6.00 c	356.29 ± 3.42 a	347.62 ± 3.41 a	9.72 ± 1.67 c	30 min	60 min/2 d	/	/
*Ulmus parvifolia*	Samara	Wind	44.28 ± 9.77 b	16.99 ± 1.31 c	340.53 ± 1.22 a	336.43 ± 4.85 a	349.11 ± 11.33 a	15 min	30 min/1 d	/	/
*Acer buergerianum*	Samara	Wind	50.30 ± 0.49 b	334.12 ± 2.31 a	331.76 ± 4.81 a	328.99 ± 1.25 a	334.15 ± 3.84 a	15 min	30 min/1 d	/	/
*Eucommia ulmoides*	Samara	Wind	49.87 ± 0.65 b	348.81 ± 6.13 a	355.92 ± 3.56 a	353.83 ± 0.60 a	357.93 ± 1.93 a	15 min	30 min/1 d	/	/

Mean ± SD within row followed by different lowercase letters indicates significant differences at p ≤ 0.05 based on an F–LSD test.

### Influence of staining on seed germination and weight

The 13 tested species had different diaspore weights and germination rates. Aster subulatus had the lightest diaspores with a 1,000-seed weight of about 0.03 g, *Q. acutissima* had the heaviest diaspore with a 100-seed weight of about 350 g.; *S. canadensis* had the lowest germination rate (about 40%) and *B. syzigachne*, the highest (about 80%). Despite this variability in seed weight and germination rate, the staining with safranine did not significantly affect either trait in any of the tested species (Table 3).

**TABLE 3:** Influence of staining seeds with safranine on seed weight and germination.

Species	1,000–seed weight (g)		Germination rate (%)	
		
	Pre-stained seeds	Stained seeds	Pre-stained seeds	Stained seeds
*Oryza sativa*	21.846 ± 0.162 ^ns^	21.933 ± 0.157 ^ns^	77.00 ± 5.87 ^ns^	76.40 ± 5.18 ^ns^
*Beckmannia syzigachne*	0.898 ± 0.020 ^ns^	0.906 ± 0.026 ^ns^	81.40 ± 3.21 ^ns^	80.20 ± 3.56 ^ns^
*Alopecurus japonicus*	0.343 ± 0.022 ^ns^	0.350 ± 0.016 ^ns^	72.00 ± 3.54 ^ns^	70.60 ± 5.86 ^ns^
*Echinochloa crusgalli*	2.042 ± 0.056 ^ns^	2.037 ± 0.121 ^ns^	72.40 ± 5.03 ^ns^	74.00 ± 5.92 ^ns^
*Phragmites communis*	0.384 ± 0.025 ^ns^	0.397 ± 0.010 ^ns^	80.00 ± 3.74 ^ns^	79.20 ± 6.06 ^ns^
*Cyperus difformis*	0.023 ± 0.001 ^ns^	0.024 ± 0.004 ^ns^	78.40 ± 7.80 ^ns^	73.40 ± 5.94 ^ns^
*Solidago canadensis*	0.045 ± 0.010 ^ns^	0.046 ± 0.009 ^ns^	39.40 ± 3.65 ^ns^	38.00 ± 4.06 ^ns^
*Aster subulatus*	0.028 ± 0.003 ^ns^	0.031 ± 0.004 ^ns^	42.80 ± 7.98 ^ns^	41.60 ± 4.39 ^ns^
*Pterocarya stenoptera*	77.871 ± 5.555 ^ns^	76.901 ± 4.817 ^ns^	74.40 ± 3.78 ^ns^	73.20 ± 4.21 ^ns^
*Quercus acutissima* *	351.672 ± 46.110 ^ns^	362.094 ± 38.188 ^ns^	38.00 ± 5.70 ^ns^	39.00 ± 7.42 ^ns^
*Ulmus parvifolia*	7.880 ± 0.361 ^ns^	7.808 ± 0.458 ^ns^	70.80 ± 7.16 ^ns^	68.40 ± 6.99 ^ns^
*Acer buergerianum*	22.462 ± 1.574 ^ns^	23.184 ± 1.665 ^ns^	67.20 ± 7.33 ^ns^	70.60 ± 5.08 ^ns^
*Eucommia ulmoides*	69.576 ± 5.128 ^ns^	71.120 ± 7.294 ^ns^	74.20 ± 5.22 ^ns^	74.00 ± 6.44 ^ns^

*100–seed weight (g). Mean ± SD of the same item within row followed by “ns” indicates no significant differences at p ≤ 0.05 based on an F–LSD test.

### Application of staining technique in dispersal experiments

The four selected species *B. syzigachne*, *O. sativa*, *S. canadensis*, and *A. buergerianum* had different dispersal modes. With the help of *ex situ* or *in situ* staining approaches by safranine, the long-distance and short-distance seed dispersal of the four species via different vectors were tracked (Table 1). For *B. syzigachne*, about 1,200,000 seeds were stained *ex situ* and used in seed release experiments. Seeds of *B. syzigachne* floated on water by virtue of their special “air bladders” and were dispersed over 2,000 m by irrigation water in ditches (Zhang et al., 2019). For *O. sativa*, about 15,000 seeds were stained *in situ* before seed shattering. After harvest, the stained weedy-rice seeds were found over 3,000 m away from its site of production, in the harvest pocket, on the pedrail, and on the metal plate of the harvester (Gao et al., 2018). More than one million seeds of *S. canadensis* were stained *in situ* which were later naturally dispersed by wind in all directions. At the prevailing wind direction, the seeds were dispersed over 2,000 m away from the parent plants. In other directions, the seed dispersal distances were about 1,000 m. After the >5,000 seeds of *A. buergerianum* stained *in situ* shattered, stained seeds were found at a distance greater than 20 m away from the parent tree. Therefore, the safranine staining can be used for studying of seed dispersal via different agents in various environments.

### *Ex situ* staining effect of plant species with safranine

**FIGURE 1:**
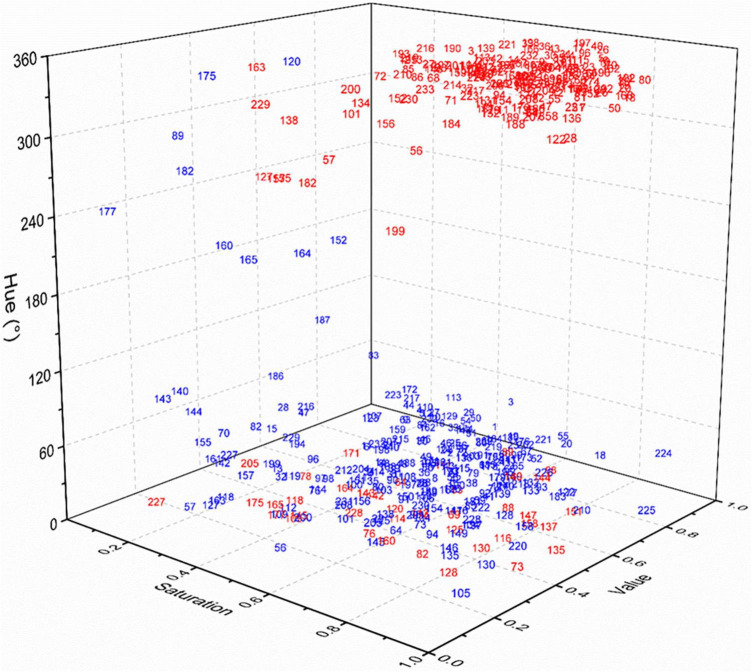
HSV color space of pre-stained and post-stained seeds/fruits. Gramineae: 1–57; Asteraceae: 58–86; Labiatae: 87–95; Euphorbiaceae: 96–108; Leguminosae: 109–120; Cyperaceae: 121–129; Brassicaceae: 130–138; Polygonaceae: 139–147; Caryophyllaceae: 148–152; Scrophulariaceae: 153–159; Convolvulaceae: 160–165; Apiaceae: 166–170; Amaranthaceae: 171–177; Others: 178–233.

Among the 233 species tested, the diaspores of 181 species, seeds of 42 species, and fruits of 139 species, belonging 159 genera in 50 families could be easily distinguished by eye from the corresponding pre-stained seeds and fruits after *ex situ* staining with 1.0% safranine (Figure 1). They were regarded as marked species, representing 77.7% of all tested species. HSV color space, which was established based on the hue, saturation, and value, showed that the 181 marked species of post-stained seeds/fruits had a hue ranging from 300 to 360° (from magenta to red). Correspondingly, the hue of pre-stained seeds/fruits of these species had the range of hue from 30 to 70° (from orange to yellow). Fruits of Gramineae and Asteraceae, and seeds of Euphorbiaceae were most easily stained, including 89 species in total, while fruits or seeds of 18 species belonging to the Cyperaceae, Labiatae, Leguminosae, and Polygonaceae were found to be more difficult to stain.

**FIGURE 2:**
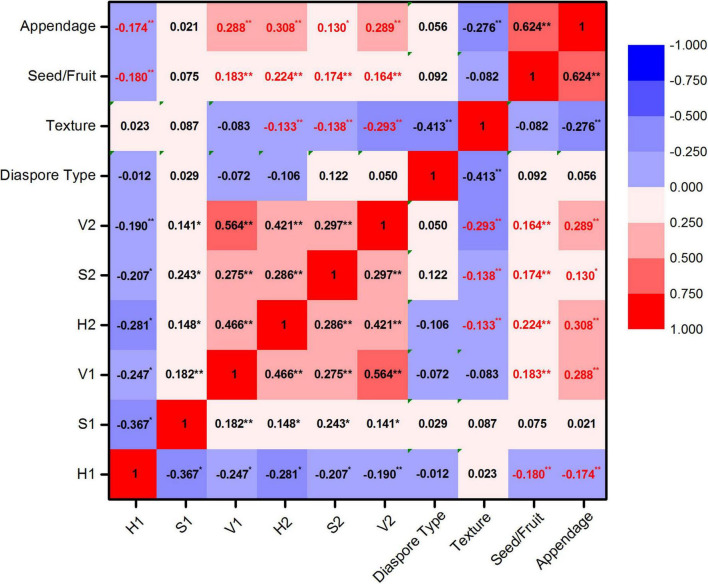
Correlation analysis of HSV color space and seeds/fruits characters. Hue, saturation, and value of diaspores before staining were represented by H1, S1, and V1 and after staining were represented by H2, S2, and V2. *Significant correlation; **highly significant correlation.

The hue of post-stained seed/fruit was significantly negatively correlated with the texture (*r* = −0.133, *p* < 0.05), but significant positively correlated with the propagule type (seed or fruit) (*r* = 0.224, *p* < 0.01) and the presence of appendages (*r* = 0.308, *p* < 0.01) (Figure 2). Fruits with appendages and fluffy texture stained more easily than seeds without appendages and with hard texture. The texture of the easy stained appendages was usually papery or scarious but nuts, utricles, legumes, follicles, siliques, berries, drupes, and cremocarps with leathery or crustaceous appendages were difficult to stain.

### Classification of staining ability of plant species

According to the source, all the 233 test species were in three categories.

#### Farmland weeds

**FIGURE 3:**
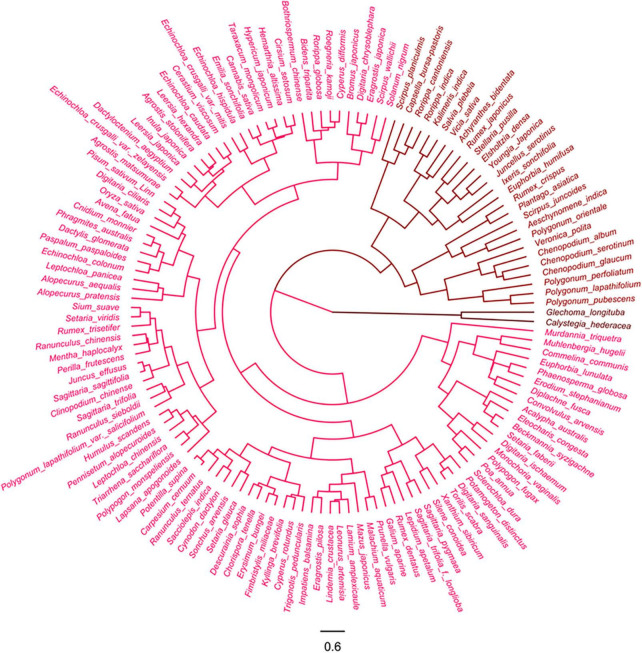
Cluster analysis of farmland weeds based on color of pre- and post-stained seeds/fruits. Red color indicates visibility of stained seeds from visible to invisible.

The 135 farmland weeds were clustered into three groups in terms of the HSV color model of their pre-stained and post-stained diaspores (Figure 3).

Group 1: This corresponded to the lighted colored episperm/pericarp or appendages of 106 species diaspores, accounting for 78.5% of all tested farmland species, which readily stained magenta to red (320–360°) from natural light color (30–60°) thus becoming easily distinguishable from the pre-stained ones. Most of the tested diaspores (78 species) in this group were fruits with appendages, including those with light-colored glumes, lemmas, and paleas or with appendages with grass-like, tissue-like, or papery textures. Most of the gramineous weeds (44 species) belong to this group (Supplementary Figure 1).

Group 2: This group included 27 species of diaspores, which was not stained effectively, resulting in the absence of differences in color between the post-stained and pre-stained diaspores. The natural and post-staining hue of these diaspores varied from red to yellow (0–60°), but the value of these diaspores was low (lower than 0.5). Half of the tested diaspores (12 species) were seeds without any appendages or the appendages fell off and had a stiff or leathery texture (Supplementary Figure 2).

Group 3: This group only included seeds of *Calystegia hederacea* and *Glechoma longituba*, lacking appendages and having naturally dark color (black and dark brown, hue between 200 and 360° and the value less than 0.5) (Supplementary Figure 3).

#### Invasive alien plants

**FIGURE 4:**
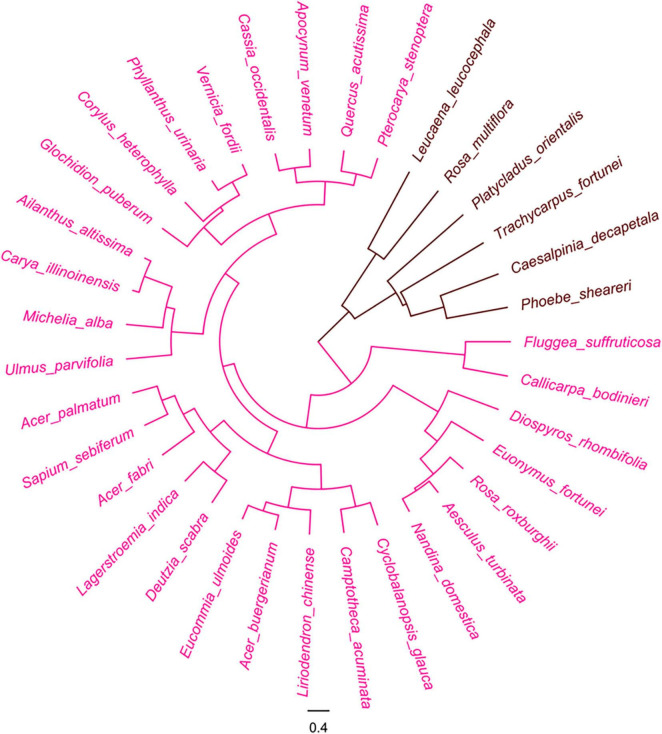
Cluster analysis of invasive plants based on the color of pre- and post-stained seeds/fruits. Red color indicates visibility of stained seeds from visible to invisible.

The 63 invasive alien plants were divided into four groups based on the hue of their diaspores according to the cluster analysis (Figure 4).

Group 1: This group included 28 species, accounting for 44.4% of all tested invasive alien species, whose diaspores with orange to mid yellow color (hue from 30 to 60°) were designated as light color group. They were stained into magenta to red (hue from 320 to 360°) of various shades. More than half of these species belong to Asteraceae (10 species) that had achenes adhered with pappi or wings at their top or side and Poaceae (9 species) whose caryopses were covered with light colored lemma and palea and tissue-like or papery glume (Supplementary Figure 4).

Group 2: This group included 18 species, accounting for 28.6% of all tested invasive alien species, whose diaspores were orange-yellow to straw-yellow color in hue (25–45°) before staining. The diaspores were stained into magenta to orange-red (330–25°), and the post-stained could also be distinguished from their untreated counterparts. Except two species, *Medicago sativa* and *Veronica arvensis*, all other stained diaspores were fruits (Supplementary Figure 5).

Group 3: The group consisted of 12 species with naturally dark-pigmented colored appendages or episperm/pericarps. Though they were not easily distinguished from unstained ones due to their deep color, stained diaspores in the group could still be identified with naked eyes through careful observation of the magenta-stained hila and appendages.

Group 4: The group had five species of seeds with black or dark brown episperm (hue between 200 and 360° and the value was less than 0.3). Due to their deep color appearance, the stained diaspores could not be distinguished from the pre-stained ones by naked eyes (Supplementary Figure 6).

#### Woody plants

**FIGURE 5:**
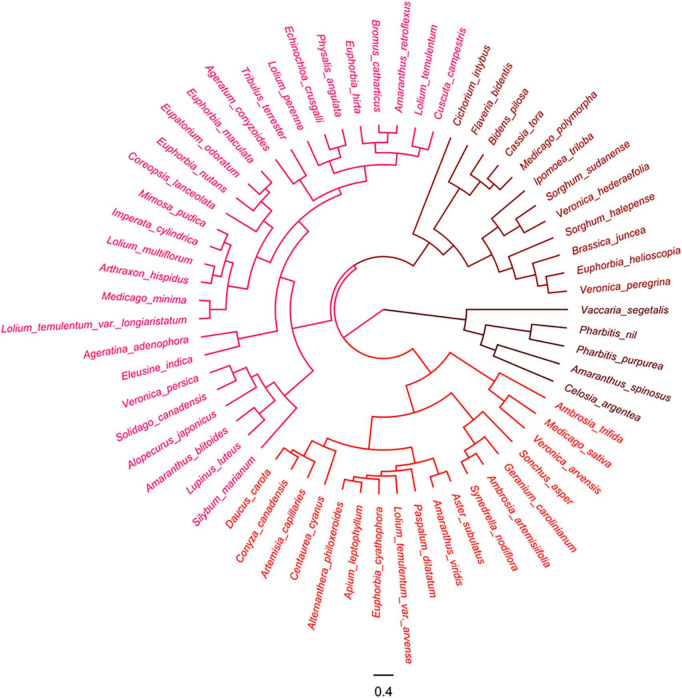
Cluster analysis of woody plants based on color of pre- and post-stained seeds. Red color indicates visibility of stained seeds from visible to invisible.

The cluster analysis also separated the 35 woody plant species into two groups based on the hue of their seeds and fruits (Figure 5).

Group 1: The group recognized as the light color group, included 29 species, accounting for 82.9% of all tested woody species, whose post-stained seeds were visibly distinguished from the pre-stained ones. The 23 species of them were fruits with wings and the remainders were the seeds included in involucres or enclosed by fruiting bracts. The appendages of all these seeds were distinctly stained by safranine (Supplementary Figure 7).

Group 2: The group of deep color diaspores included 6 species that were difficult to separate with the safranine staining. All lacked appendages and had dark color episperm (mostly kermesinus, dark brown, and black brown) (Supplementary Figure 8).

### Persistence of stained mark

**FIGURE 6:**
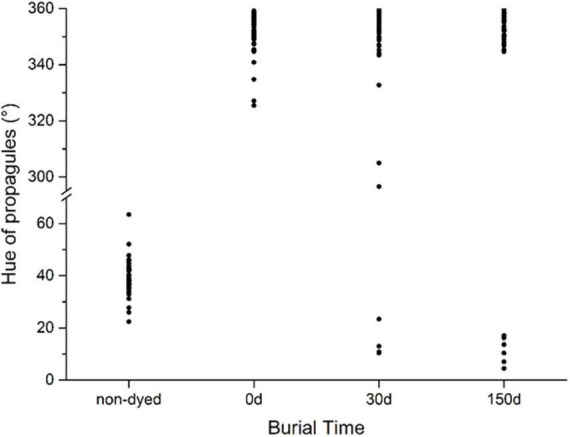
Color fading of stained diaspores buried in soil for 5 months. The hue of stained diaspores between 300 and 360° (magenta to red) indicates the color keeps identifiable; otherwise, the color fades.

The diaspores of selected 48 species were stained and buried into soil for 5 months. Before staining, the hues of these diaspores were yellow (hue between 30 and 70°) in various shades; after staining, the hues of all diaspores changed to magenta to red (hue between 320 and 360°) in various shades. After burial in soil for 1 month, the magenta colors of diaspores from 44 weed species, representing 97% of all buried species, were still visible and easily distinguished from unstained ones. Only four species faded, including two seeds *Silene conoidea*, *Malachium aquaticum* and two fruits with the bract fell off *Rumex japonicas* and *Kyllinga brevifoli*. After burial in soil for 5 months, the seed color of another two stained species (*Calypha australis* and *Setaria faberii*) faded (Figure 6).

### Quick identification and count of stained diaspores

Based on the image processing using OpenCV and Numpy on Python platform, the safranine-stained diaspores can be distinguished from background and unstained diaspores and counted on a computer quickly and efficiently. In the stained group and mixed group, the stained diaspores were exactly counted with no counting error in 85% (115) and 76% (103) of the 136 tested photographs, respectively. While in the unstained group, diaspores in 70% (95) of the tested 136 photographs were counted correctly. Comparing the stained group with the unstained group, the count correct rate was greatly improved by staining. In mixed group, the staining marked diaspores could be effectively distinguished from unstained ones and then counted correctly.

## Discussion

### Seed staining and seed structure

The stainability of diaspores (seeds or fruits) mainly depends on their color because the HSV of safranine itself is fixed. Our test results showed that over 77.7% of all tested diaspores (78.5% of farmland weeds, 73.1% of invasion plants, and 82.9% of woody plants, respectively) were the greenish-yellow to orange diaspores (hue from 30 to 70°) which is easily stained into magenta to red color (hue from 320 to 360°) by 1% safranine. The most easily distinguished diaspores after staining are naturally light-colored. 16.7% of the remainder with dark-colored diaspores can still be distinguished if they have light-colored structures (e.g., umbilici). However, 5.6% of very dark diaspores lacking any light-colored structures were unsuitable for safranine staining. Totally, these stainable and distinguishable diaspores occupied 94.4% of all the 233 tested species. Therefore, regardless of the light-colored part is pericarp, seed coat, or appendage, the diaspores, which have the light-colored part, may be stainable and stained diaspores may be visibly distinguishable. Furthermore, the magenta color of the safranine is strikingly different from the color of the majority of plant diaspores in nature, which contributes to the differentiation of the stained diaspores.

The staining effect of diaspores is influenced by the texture of their episperm/pericarp covered or/and appendages attached. The diaspores with fluffy texture, which can adsorb in safranine readily and combine the stain firmly, can be usually stained more easily. In contrast, the diaspores were leathery, stiff, and smooth-faced, and without appendages usually poorly adsorb the dyestuff or somewhat easily remove the adsorbed dyestuff, resulting in difficult staining. In addition, appendages of diaspores are easily stained due to their fluffy texture and light color. However, compared to the fruits, seeds usually lack pericarps with more fluffy texture and do not have various appendages, resulting in more difficult staining. In this study, 86.3% of tested fruits can be effectively stained and easily distinguished, whereas only 58.3% of tested seeds. Besides, the remainder of 13.7% of tested fruits and 23.6% of the remaining 41.7% of tested seeds can still be identified by naked eyes carefully. From the types, structures and colors of seeds or fruits and their appendages of the above stainable species, it can be inferred and estimated that about 90% of the species can be stained in the seed plants. In general, the safranine staining technique is broad-spectrum making it suitable for marking multifarious light-colored diaspores, especially those with fluffy texture, from a variety of species that live in different environments, such as farmland, wasteland, swap, and forests.

### Application of the safranine stain technique

In this study, two staining methods by safranine were applied, *in situ* staining and *ex situ* staining. The two methods were different in their operation approaches but provided similar and good staining effect for both invasion and native plants. Both staining methods can be used in the research of seed dispersal and seed bank dynamics, just considering the diaspore characteristics and based on different usage scenarios. Compared with *ex situ* staining, the *in situ* staining shortened the operation time, which could be beneficial in large-scale experiments under natural conditions while the *ex situ* staining could be more suitable in simulation experiments.

For staining by safranine has no influence on diaspores weight, it indicated that the wind dispersal progress of plant diaspores, which is usually sensitive to weight, would not be affected by safranine staining. In this case, the application of the safranine staining technique would make it possible to obtain relative authentic results in the study of wind seed dispersal. In our experiment, for the study of wind dispersal (*S. canadensis* and *A. buergerianum)*, the *in situ* staining method by safranine was successfully applied to mark the seeds. By seeking the stained plant diaspores, the dispersal data was obtained and their short and long distance dispersal was demonstrated directly. For staining by safranine has no influence on floating ability (Zhang et al., 2019), and the color of stained seeds/fruit can be retained in the water environment. Therefore, this stain technique can be used in the study of water seed dispersal. In our previous study (Zhang et al., 2019), the *ex situ* stain method was successfully used in several simulative water dispersal experiments of a noxious weed, *B. syzigachne.* By tracking the movement of stained floating seeds, the dispersal pattern was determined. In addition, the *in situ* staining method by safranine was also successfully used to mark the seeds of a malignant farmland weed, weedy rice, for the study of their dispersal by agricultural machinery (Gao et al., 2018). All the above experiments directly indicated that the seed staining method by safranine can be used in the study of long-distance and short-distance seed dispersal via wind, water, and machinery in both simulated environments and natural environments.

Furthermore, it has been demonstrated in this study that the color of stained plant seeds/fruits can still be identified when buried in soil for 5 months; the color of some stained diaspores can even last for more than 1 year (unpublished data). In addition, staining by safranine has no influence on seed germination. As a result, the stain technique can be used in the quantitative research of plant life cycle, especially the population dynamics, the seed bank dynamics, and seedling emergency. With the *in situ* staining of weed seeds during the mature stage, the seeds of the target weed can be visually marked; further, the seed shattering and the newly formed seed bank can be quantified. Moreover, the spatial distribution of seeds in soil and the change of seed bank with time can also be evaluated by appropriate soil sampling. The *in situ* staining technique has already been used in the study of the seed bank dynamics of weedy rice as we had expected (Zhang et al., 2019). Because of its safety, the stain technique can also be used in the survival strategy research of rare species which would provide useful information for the protection of such species.

### Identification and count of stained diaspores

After successful marking of target diaspores by safranine staining technique at the beginning, the identification and count of staining marked diaspores in retrieved samples could be the key link in the subsequent processes. Usually, the retrieved samples are mixed with stained and unstained diaspores and the stained diaspores need to be distinguished from unstained ones under microscope and then counted. The count of the stained ones by naked eyes in such mixture would be time-consuming, especially for the disapores with small size. In this study, the qualitative analysis by computer program was considered for quick identification and count of target stained diaspores. When the samples of diaspores were photographed, based on the striking magenta color of stained diaspores, the qualitative analysis of stained diaspores by computer program was realized by using the computer vision library OpenCV with python code with acceptable count correct rate (>75%). With the adjustment of parameters, especially the morphological process methods, the count accuracy could be improved. As a result, the identification and count of stained diaspores by computer is recommended for its high efficiency which is time and labor saving. On the one hand, this indicates an alternative sampling method by taking a photograph of target diaspores in place in field research, instead of bringing them back to the laboratory.

### Advantages and disadvantages of the staining technique

Diaspore staining with safranine has some obvious advantages when compared with other marking methods, such as isotope-labeling (e.g., Carlo et al., 2009; Forster and Herrmann, 2014), molecular techniques (e.g., Godoy and Jordano, 2001; García et al., 2007).

First, the staining method is cost-effective and simple to conduct. The safranine staining solution is simple to prepare, stable, and does not require expensive equipment or dangerous solvents. The composite cost for 10 L staining solution is about $70 ($50 for 100 g safranine, $20 for 5 L ethanol). This solution can be used to stain thousands or even millions of seeds/fruits. The staining method is easy to carry out and the staining time is short, including the drying time. Only 30 min and 24 h are required for *in situ* and *ex situ* staining, respectively. For molecular marker techniques, DNA primers are needed for the study species. Currently, primers are only available for a small group of species and taxonomic groups. Although the cost of primer development is decreasing rapidly, current costs for the development of primers in novel systems range from U.S. $3 000 to over $15 000, and development requires 3–12 months of skilled lab work. Isotope marking demands months for sample preparation and costs about U.S. $900 in equipment and mass spectrometry (Carlo et al., 2009). Comparatively speaking, stain by safranine represents a substantial saving in time and money.

Second, the stain method may have less limitation to various seed sizes and seed types. In general, the staining method by safranine described in this study can be used to mark a variety of plant diaspores, provided that they have light-colored structures. In contrast, molecular marker techniques are most suitable for large seeds with abundant endosperm (Godoy and Jordano, 2001; Khodwekar et al., 2015), telemetric thread tags need relatively large seeds and the dispersers are limited (Hirsch et al., 2012b).

Third, safranine is environmentally safe with lasting staining effect. While the radioisotope labeling is environmentally unfriendly, seed-tagging methods would affect seed survival (Xiao et al., 2006), the color powder and fluorescent color labeling fade easily or can be easily washed off (Lemke et al., 2009). The safranine is a natural dyestuff derived from the stigma of saffron. Staining by safranine has no biological harm to the seed/fruit itself and the color of stained seed/fruit can last for a relatively long period, more than 6 months, under dry and buried conditions. Even steeped in water, the color can also maintain for more than 7 days.

There are also some limits that should be emphasized. When using the *in situ* staining technique, it is important to consider the phenological phases of the plant species. It is best to implement the *in situ* staining method at seed maturity stage under dry weather conditions. When the staining method is used, regardless of *in situ* or *ex situ*, the quantity of plant diaspores should be taken into consideration for the stained quantity can influence the accuracy and reliability of study results. If used to stain a small amount of diaspores and without a suitable sampling method, not finding the stained ones at later times would invalidate the experiments, especially in the study of long-distance seed dispersal.

## Conclusion

The safranine staining technique described in this study is novel, simple, environmentally friendly, inexpensive, and effective, and it can be widely used to stain light-colored or some dark-colored fruits and seeds. The specially complied Python-based program executing identification and count of the stained seeds/fruits on a computer may make investigation quick and convenient. The marking seed method may be a valuable tool for studies of seed dispersal, seed bank dynamics, and forest succession and recruitment under various ecosystems. In future, the staining technique, including the formulation (e.g., staining agent, auxiliaries, solvents) and treatment procedure could be improved for a better and more lasting staining effect. Furthermore, the corresponding monitoring, identification, and retrieving method of staining marked diaspores under different scenarios should be studied to provide guidance on the technique application in practice for more researchers.

## Data availability statement

The original contributions presented in this study are included in the article/Supplementary material. Further inquiries can be directed to the corresponding author.

## Author contributions

ZZ and SQ conceived and designed the research. ZZ, XS, and RL collected the data. ZZ performed the analysis and drafted the manuscript. All authors provided manuscript modifications and gave approval for publication.

## Footnotes

1
http://opencv.org

2
https://www.r-project.org/

3
http://tree.bio.ed.ac.uk/software/figtree/
