# Supplementary material for: A new technique for stain-marking of seeds with safranine to track seed dispersal and seed bank dynamics
Source: Front Plant Sci. 2022 Aug 8;13:959046. doi: 10.3389/fpls.2022.959046 (PMC9393530; doi:10.3389/fpls.2022.959046)
Supplement: Supplementary file 1 [file Data_Sheet_1.docx]

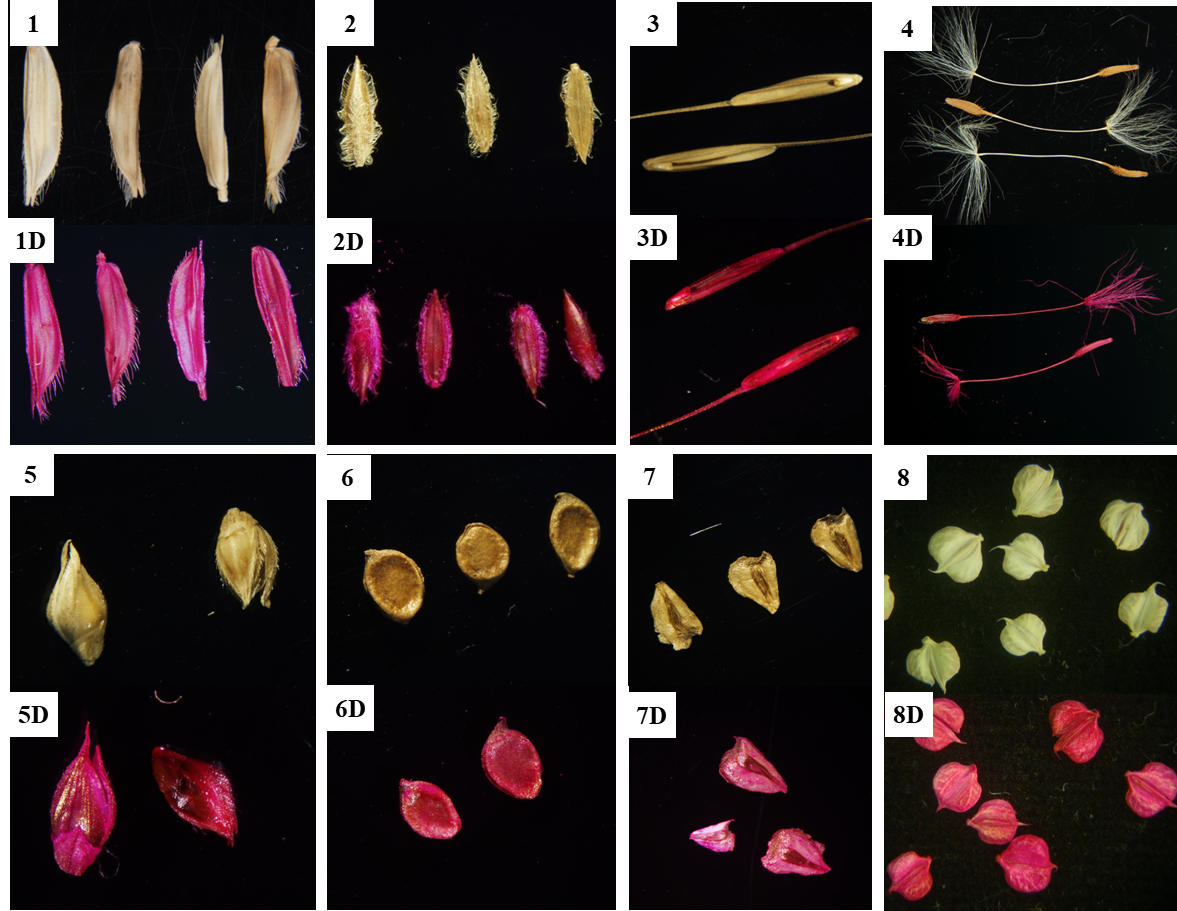


Figure 1 Some diaspores of farmland weeds for which distinguishing between post-stained and pre-stained fruits or seeds is easy; Upper diaspores showing their natural color; bottom diaspores after staining with safranine; Species: (1)*Leersia japonica*; (2)*Digitaria ciliaris*; (3)*Roegneria kamoji*; (4)*Taraxacum mongolicum*; (5)*Echinochloa crusgalli* (L.) Beauv.; (6)*Ranunculus chinensis*; (7)*Sagittaria trifolia*; and (8)*Beckmannia syzigachne.*


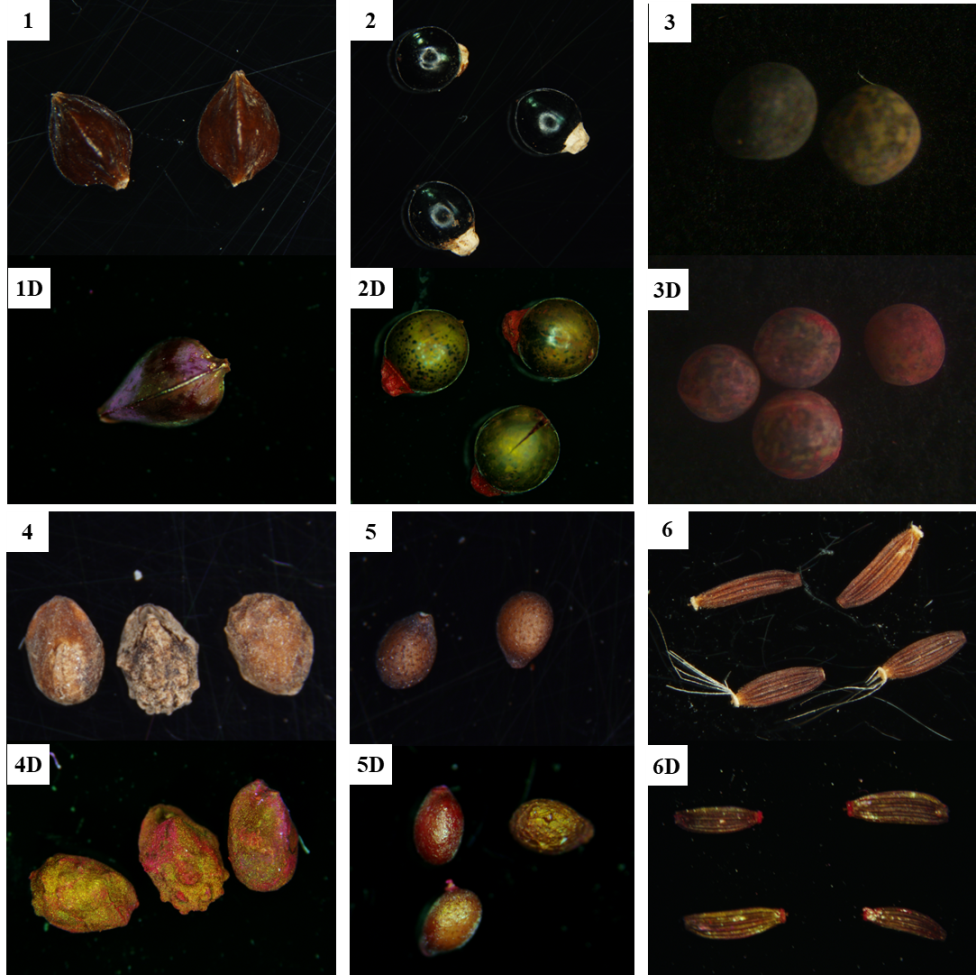


Figure 2 Some diaspores of farmland seeds/fruits for which distinguishing between post-stained and pre-stained fruits and seed is a little difficult; Upper diaspores showing their natural color; bottom diaspores after staining with safranine; Species: (1) *Rumex crispus*; (2)*Polygonum perfoliatum* ; and (3)*Vicia vicia*; (4)*Elsholtzia densa*;(5) *Salvia plebeia*; (6)*Youngia Japonica*


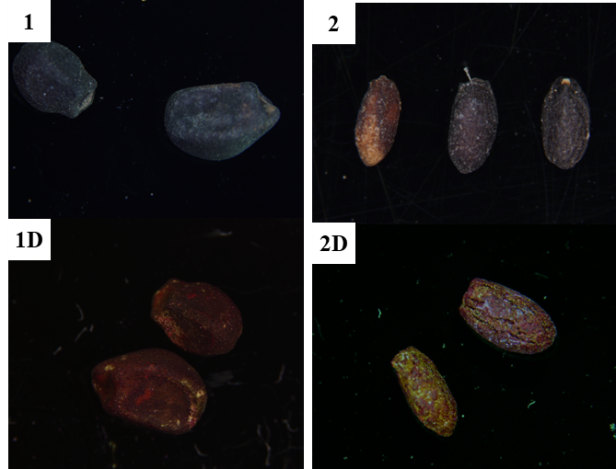


Figure 3 Diaspores of farmland weeds for which distinguishing between post-stained and pre-stained fruits and seed is difficult; Upper diaspores showing their natural color; bottom diaspores after staining with safranine; Species: (1)*Calystegia hederacea*; and (2)*Glechoma longituba*


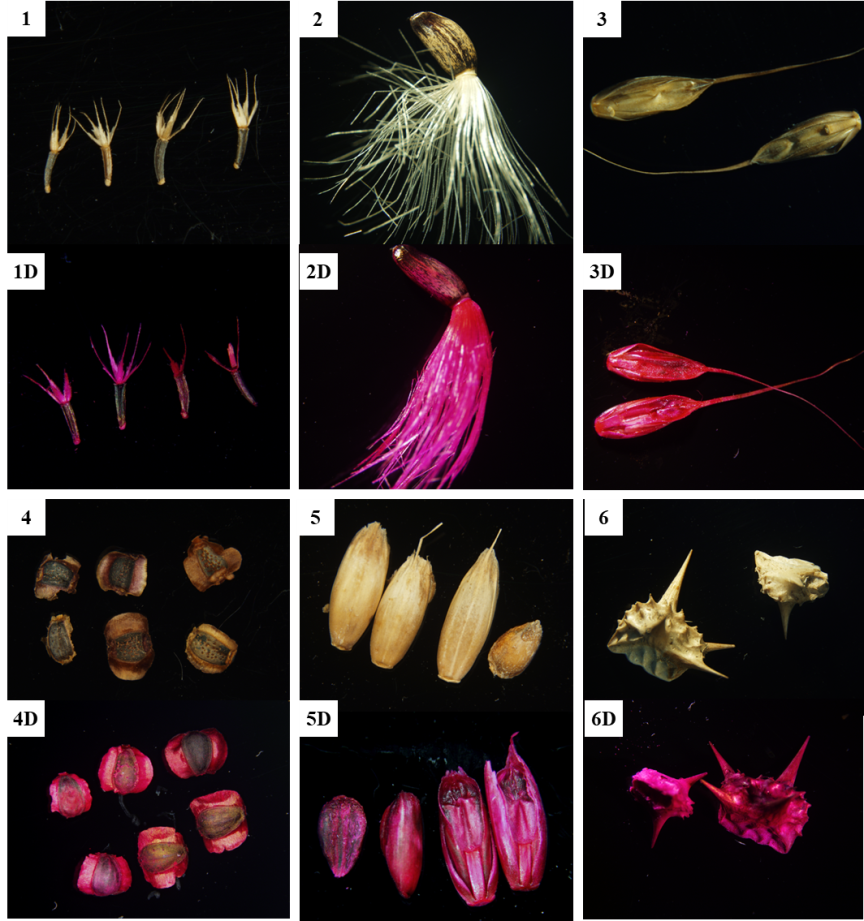


Figure 4 Some diaspores of invasive alien plants which distinguishing between post-stained and pre-stained fruits and seed is easy; Upper diaspores showing their natural color; bottom diaspores after staining with safranine; Species: (1)*Ageratum conyzoides*; (2)*Silybum marianum*; (3)*Lolium temulentum* L. var. *longiaristatum Parnell*; (4)*Coreopsis lanceolata*; (5)*Lolium temulentum* var. arvense Bab*.*; and (6)*Tribulus terrester*.


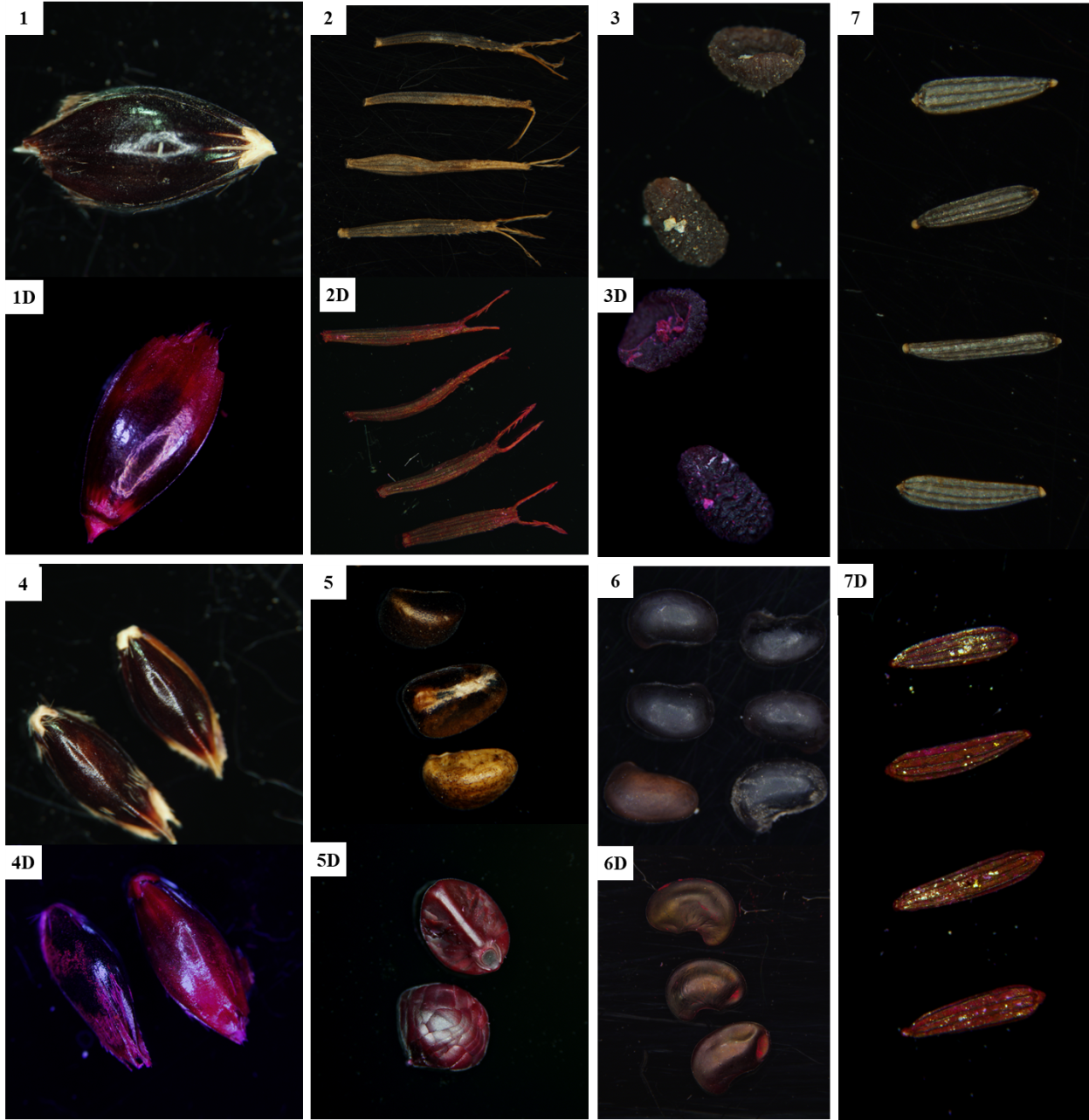


Figure 5 Diaspores of invasive alien plants for which distinguishing between post-stained and pre-stained fruits and seed is a little difficult. Upper diaspores showing their natural color; bottom diaspores after staining with safranine. Species: (1)*Sorghum sudanense*, (2)*Bidens pilosa*; (3)*Veronica hederaefolia*; (4)*Sorghum halepense*; (5)*Ipomoea triloba*; (6)*Aeschynomene indica*, and (7)*Flaveria bidentis*.


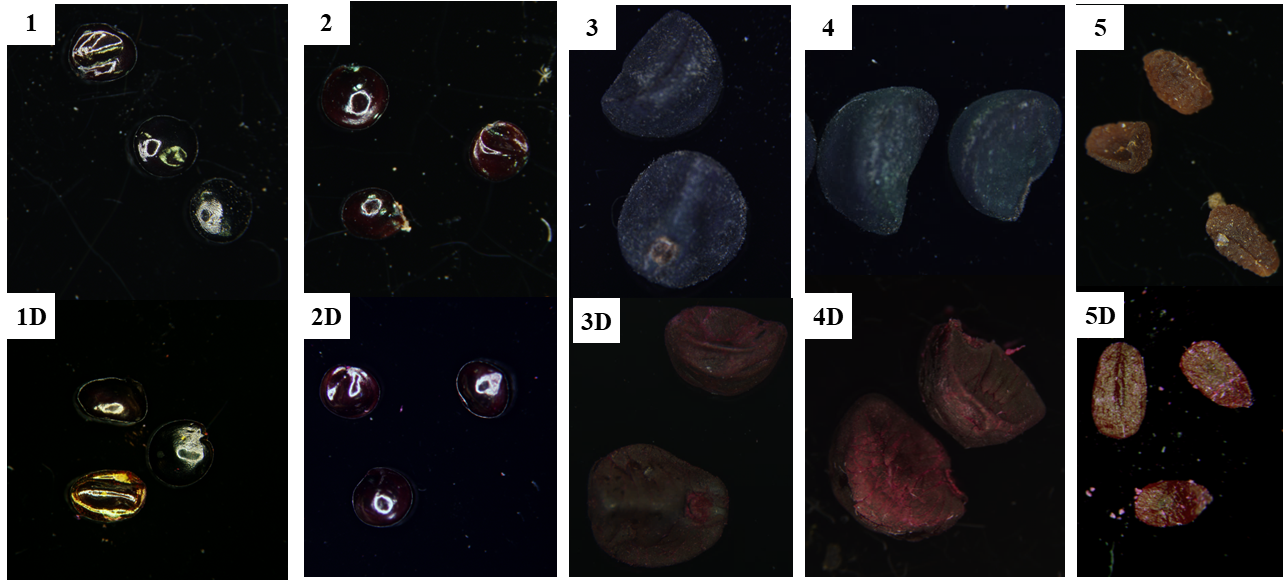


Figure 6 Diaspores of invasive alien plants for which distinguishing between post-stained and pre-stained fruits and seed is difficult; Upper diaspores showing their natural color; bottom diaspores after staining with safranine; Species: (1)*Celosia argentea*; (2)*Amaranthus spinosus*; (3)*Pharbitis purpurea*; (4)*Pharbitis nil* and (5)*Veronica peregrina*


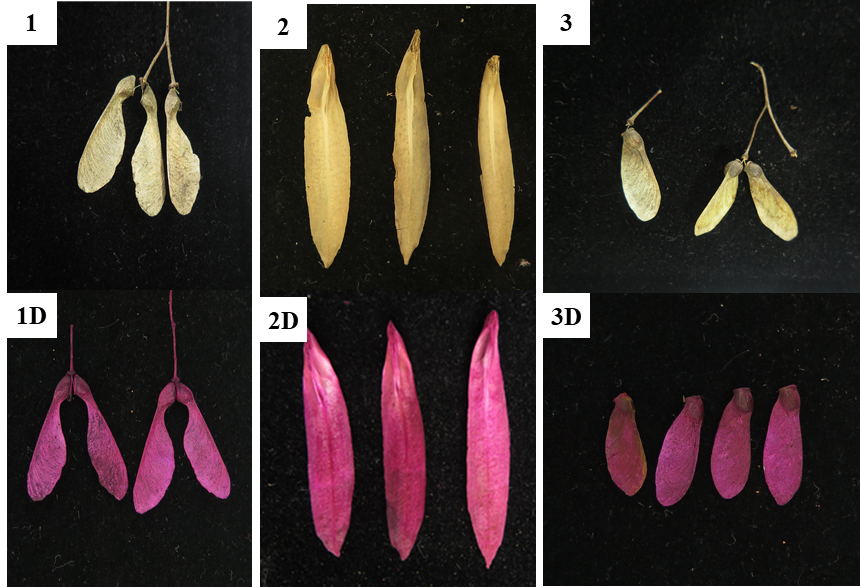


Figure 7 Diaspores of woody plants for which distinguishing between post-stained and pre-stained fruits and seed is easy; Upper diaspores showing their natural color; bottom diaspores after staining with safranine; Species: (1)*Acer buergerianum*; (2)*Liriodendron chinense*; and (3)A*cer palmatum*.


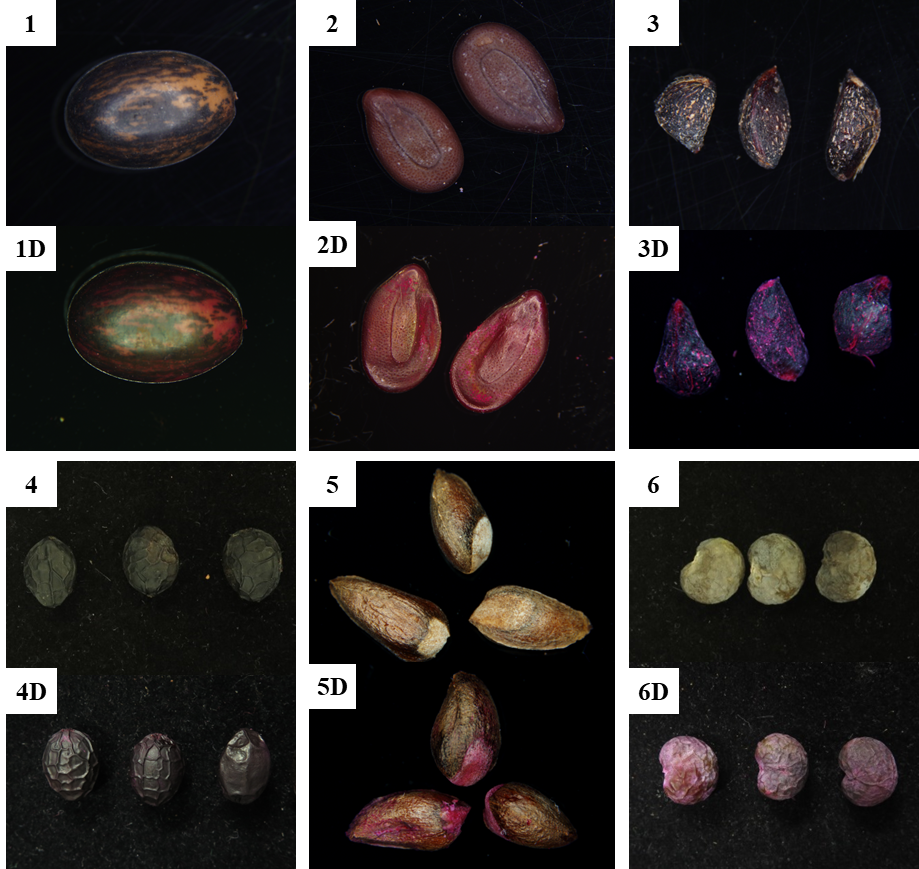


Figure 8 Diaspores of woody plants for which distinguishing between post-stained and pre-stained fruits and seed is difficult; Upper diaspores showing their natural color; bottom diaspores after staining with safranine; Species: (1)*Caesalpinia decapetala*; (2)*Leucaena leucocephala*; (3)*Rosa multiflora*; (4)*Phoebe sheareri*; (5)*Platycladus orientalis*; and (6)*Trachycarpus fortune*.
