# Supplementary material for: A new technique for stain-marking of seeds with safranine to track seed dispersal and seed bank dynamics
Source: Front Plant Sci. 2022 Aug 8;13:959046. doi: 10.3389/fpls.2022.959046 (PMC9393530; doi:10.3389/fpls.2022.959046)
Supplement: Supplementary file 2 [file Data_Sheet_2.docx]

Table 1 HSV color space of pre-stained and stained seeds/fruits of farmland plants

| Serial number | Family | Latin Name | Seed type | Unstained | | | Stained | | | Seed/  Fruit | Appendage |
| --- | --- | --- | --- | --- | --- | --- | --- | --- | --- | --- | --- |
|  |  |  |  | H1 | S1 | V1 | H2 | S2 | V2 |  |  |
| 1 | Poaceae | *Agrostis matsumurae* | Caryopsis | 40.18 | 0.53 | 0.81 | 338.98 | 0.94 | 0.65 | Fruit | Lemma and palea |
| 2 |  | *Agrostis stolonifera* | Caryopsis | 40.69 | 0.56 | 0.61 | 346.90 | 0.87 | 0.64 | Fruit | Lemma and palea |
| 3 |  | *Alopecurus aequalis* | Caryopsis | 52.12 | 0.51 | 0.90 | 359.05 | 0.60 | 0.62 | Fruit | Lemma |
| 4 |  | *Alopecurus pratensis* | Caryopsis | 50.57 | 0.38 | 0.73 | 336.36 | 0.52 | 0.74 | Fruit | Lemma |
| 5 |  | *Avena fatua* | Caryopsis | 38.99 | 0.34 | 0.78 | 345.44 | 0.72 | 0.91 | Fruit | Lemma |
| 6 |  | *Beckmannia syzigachne* | Caryopsis | 45.79 | 0.42 | 0.49 | 348.99 | 0.78 | 0.85 | Fruit | Lemma |
| 7 |  | *Bromus japonicus* | Caryopsis | 41.54 | 0.45 | 0.56 | 331.53 | 0.90 | 0.60 | Fruit | Lemma and palea |
| 8 |  | *Cynodon dactylon* | Caryopsis | 35.84 | 0.61 | 0.50 | 319.51 | 0.79 | 0.61 | Fruit | Lemma and palea |
| 9 |  | *Dactylis glomerata* | Caryopsis | 50.00 | 0.39 | 0.66 | 343.31 | 0.63 | 0.82 | Fruit | Lemma and palea |
| 10 |  | *Dactyloctenium aegyptium* | Caryopsis | 48.31 | 0.42 | 0.72 | 355.50 | 0.85 | 0.74 | Fruit | Lemma and palea |
| 11 |  | *Digitaria chrysoblephara* | Caryopsis | 31.20 | 0.34 | 0.58 | 325.63 | 0.74 | 0.55 | Fruit | Lemma |
| 12 |  | *Digitaria ciliaris* | Caryopsis | 47.50 | 0.39 | 0.73 | 333.58 | 0.78 | 0.67 | Fruit | Lemma |
| 13 |  | *Digitaria ischaemum* | Caryopsis | 31.20 | 0.29 | 0.34 | 350.16 | 0.91 | 0.55 | Fruit | Lemma |
| 14 |  | *Digitaria sanguinalis* | Caryopsis | 42.27 | 0.48 | 0.45 | 350.91 | 0.65 | 0.70 | Fruit | Lemma |
| 15 |  | *Diplachne fusca* | Caryopsis | 45.00 | 0.18 | 0.44 | 335.61 | 0.71 | 0.68 | Fruit | Lemma and palea |
| 16 |  | *Echinochloa caudata* | Caryopsis | 47.25 | 0.45 | 0.70 | 350.81 | 0.85 | 0.57 | Fruit | Lemma and palea |
| 17 |  | *Echinochloa colonum* | Caryopsis | 42.58 | 0.46 | 0.61 | 355.32 | 0.71 | 0.85 | Fruit | Lemma and palea |
| 18 |  | *Echinochloa crusgalli* var. *mitis* | Caryopsis | 41.20 | 0.81 | 0.80 | 332.09 | 0.91 | 0.74 | Fruit | Lemma and palea |
| 19 |  | *Echinochloa crusgalli* var. *zelayensis* | Caryopsis | 42.20 | 0.60 | 0.77 | 347.17 | 0.95 | 0.65 | Fruit | Lemma and palea |
| 20 |  | *Echinochloa hispidula* | Caryopsis | 42.57 | 0.72 | 0.81 | 345.66 | 0.96 | 0.65 | Fruit | Lemma and palea |
| 21 |  | *Eragrostis japonica* | Caryopsis | 34.58 | 0.39 | 0.59 | 324.83 | 0.79 | 0.58 | Fruit | Lemma and palea |
| 22 |  | *Eragrostis pilosa* | Caryopsis | 21.25 | 0.59 | 0.32 | 335.24 | 0.91 | 0.55 | Fruit | Lemma |
| 23 |  | *Hemarthria altissima* | Caryopsis | 37.12 | 0.61 | 0.76 | 349.48 | 0.80 | 0.75 | Fruit | Lemma and palea |
| 24 |  | *Leersia hexandra* | Caryopsis | 37.86 | 0.52 | 0.63 | 355.04 | 0.80 | 0.65 | Fruit | Lemma and palea |
| 25 |  | *Leersia japonica* | Caryopsis | 34.09 | 0.49 | 0.70 | 334.18 | 0.85 | 0.73 | Fruit | Lemma and palea |
| 26 |  | *Leptochloa chinensis* | Caryopsis | 38.21 | 0.61 | 0.44 | 358.55 | 0.79 | 0.82 | Fruit | Lemma |
| 27 |  | *Leptochloa panicea* | Caryopsis | 43.78 | 0.38 | 0.77 | 354.40 | 0.59 | 0.50 | Fruit | Lemma and palea |
| 28 |  | *Muhlenbergia hugelii* | Caryopsis | 60.00 | 0.18 | 0.48 | 324.11 | 0.99 | 0.42 | Fruit | Lemma |
| 29 |  | *Oryza sativa* | Caryopsis | 37.47 | 0.40 | 0.86 | 345.13 | 0.70 | 0.75 | Fruit | Lemma and palea |
| 30 |  | *Paspalum paspaloides* | Caryopsis | 43.60 | 0.47 | 0.61 | 355.41 | 0.72 | 0.74 | Fruit | Lemma and palea |
| 31 |  | *Pennisetum alopecuroides* | Caryopsis | 35.22 | 0.52 | 0.46 | 352.19 | 0.70 | 0.82 | Fruit | Lemma and palea |
| 32 |  | *Phaenosperma globosa* | Caryopsis | 30.00 | 0.32 | 0.32 | 342.56 | 0.70 | 0.48 | Fruit | Lemma and palea |
| 33 |  | *Phragmites australis* | Caryopsis | 46.21 | 0.48 | 0.71 | 356.16 | 0.60 | 0.82 | Fruit | Palea |
| 34 |  | *Poa annua* | Caryopsis | 42.24 | 0.50 | 0.40 | 349.39 | 0.86 | 0.66 | Fruit | Lemma and palea |
| 35 |  | *Polypogon fugax* | Caryopsis | 42.70 | 0.55 | 0.36 | 356.93 | 0.76 | 0.70 | Fruit | Lemma |
| 36 |  | *Polypogon monspeliensis* | Caryopsis | 37.65 | 0.57 | 0.50 | 353.33 | 0.62 | 0.85 | Fruit | Lemma |
| 37 |  | *Roegneria kamoji* | Caryopsis | 42.39 | 0.57 | 0.64 | 351.68 | 0.88 | 0.45 | Fruit | Lemma and palea |
| 38 |  | *Sacciolepis indica* | Caryopsis | 36.67 | 0.68 | 0.52 | 351.43 | 0.85 | 0.62 | Fruit | Lemma and palea |
| 39 |  | *Sclerochloa dura* | Caryopsis | 42.23 | 0.53 | 0.45 | 347.56 | 0.93 | 0.69 | Fruit | Lemma |
| 40 |  | *Setaria faberii* | Caryopsis | 47.77 | 0.47 | 0.51 | 357.45 | 0.74 | 0.87 | Fruit | Lemma and palea |
| 41 |  | *Setaria glauca* | Caryopsis | 35.48 | 0.75 | 0.49 | 345.00 | 0.90 | 0.75 | Fruit | Lemma and palea |
| 42 |  | *Setaria viridis* | Caryopsis | 36.43 | 0.64 | 0.51 | 355.47 | 0.66 | 0.63 | Fruit | Lemma and palea |
| 43 |  | *Triarrhena sacchariflora* | Caryopsis | 34.86 | 0.58 | 0.57 | 354.43 | 0.66 | 0.82 | Fruit | Lemma and palea |
| 58 |  | *Bidens tripartita* | Achene | 37.59 | 0.63 | 0.51 | 335.09 | 0.93 | 0.45 | Fruit | Pappi |
| 59 | Asteraceae | *Carpesium cernuum* | Achene | 38.77 | 0.76 | 0.44 | 353.25 | 0.82 | 0.67 | Fruit | No |
| 60 |  | *Cirsium setosum* | Achene | 40.71 | 0.65 | 0.68 | 340.27 | 0.79 | 0.74 | Fruit | Pappi |
| 61 |  | *Emilia sonchifolia* | Achene | 36.24 | 0.64 | 0.62 | 357.37 | 0.81 | 0.66 | Fruit | Pappi |
| 62 |  | *Inula japonica* | Achene | 41.54 | 0.57 | 0.71 | 349.38 | 0.84 | 0.61 | Fruit | Pappi |
| 63 |  | *Ixeris sonchifolia* | Achene | 43.81 | 0.36 | 0.69 | 6.52 | 0.56 | 0.64 | Fruit | Pappi |
| 64 |  | *Kalimeris indica* | Achene | 20.40 | 0.66 | 0.30 | 25.45 | 0.52 | 0.49 | Fruit | Pappi |
| 65 |  | *Lapsana apogonoides* | Achene | 37.95 | 0.70 | 0.65 | 338.01 | 0.74 | 0.85 | Fruit | No |
| 66 |  | *Sonchus arvensis* | Achene | 37.50 | 0.70 | 0.63 | 336.87 | 0.93 | 0.70 | Fruit | Pappi |
| 67 |  | *Taraxacum mongolicum* | Achene | 35.04 | 0.65 | 0.76 | 350.20 | 0.85 | 0.67 | Fruit | Pappi |
| 68 |  | *Xanthium sibiricum* | Achene | 34.65 | 0.60 | 0.47 | 345.79 | 0.61 | 0.49 | Fruit | Hooked spines |
| 69 |  | *Youngia Japonica* | Achene | 21.60 | 0.59 | 0.50 | 31.20 | 0.75 | 0.39 | Fruit | Pappi |
| 87 | Labiatae | *Clinopodium chinense* | Nutlet | 28.97 | 0.58 | 0.59 | 350.30 | 0.70 | 0.55 | Fruit | Calyx |
| 88 |  | *Elsholtzia densa* | Nutlet | 50.00 | 0.28 | 0.42 | 29.71 | 0.80 | 0.50 | Seed | No |
| 89 |  | *Glechoma longituba* | Nutlet | 300.00 | 0.18 | 0.17 | 44.48 | 0.67 | 0.68 | Seed | No |
| 90 |  | *Lamium amplexicaule* | Nutlet | 37.08 | 0.55 | 0.42 | 356.12 | 0.82 | 0.50 | Seed | No |
| 91 |  | *Leonurus artemisia* | Nutlet | 37.84 | 0.64 | 0.35 | 347.44 | 0.81 | 0.51 | Seed | No |
| 92 |  | *Mentha haplocalyx* | Nutlet | 25.98 | 0.69 | 0.55 | 348.00 | 0.68 | 0.60 | Seed | No |
| 93 |  | *Perilla frutescens* | Nutlet | 31.68 | 0.79 | 0.62 | 351.27 | 0.68 | 0.64 | Seed | No |
| 94 |  | *Prunella vulgaris* | Nutlet | 34.01 | 0.78 | 0.26 | 338.28 | 0.75 | 0.52 | Seed | No |
| 95 |  | *Salvia plebeia* | Nutlet | 12.73 | 0.60 | 0.43 | 4.86 | 0.59 | 0.49 | Seed | No |
| 96 | Euphorbiaceae | *Acalypha australis* | Capsule | 46.14 | 0.38 | 0.35 | 351.27 | 0.71 | 0.87 | Seed | No |
| 97 |  | *Euphorbia lunulata* | Capsule | 6.86 | 0.28 | 0.49 | 352.50 | 0.77 | 0.65 | Seed | No |
| 98 |  | *Euphorbia humifusa* | Capsule | 21.01 | 0.36 | 0.42 | 10.83 | 0.66 | 0.85 | Seed | No |
| 109 | Leguminosae | *Aeschynomene indica* | Pod | 7.50 | 0.35 | 0.27 | 12.86 | 0.43 | 0.25 | Seed | No |
| 110 |  | *Pisum sativum* | Pod | 45.67 | 0.34 | 0.77 | 330.41 | 0.75 | 0.76 | Seed | No |
| 111 |  | *Vicia sativa* | Pod | 39.23 | 0.50 | 0.38 | 11.29 | 0.46 | 0.71 | Seed | No |
| 121 | Cyperaceae | *Cyperus difformis* | Netlet | 38.32 | 0.56 | 0.56 | 359.33 | 0.86 | 0.49 | Fruit | Glume |
| 122 |  | *Cyperus rotundus* | Netlet | 33.66 | 0.67 | 0.48 | 355.00 | 0.72 | 0.52 | Fruit | No |
| 123 |  | *Eleocharis congesta* | Netlet | 72.69 | 0.43 | 0.47 | 341.35 | 0.74 | 0.78 | Fruit | Glume |
| 124 |  | *Fimbristylis miliaceae* | Netlet | 28.85 | 0.70 | 0.58 | 340.73 | 0.81 | 0.53 | Fruit | Glume |
| 125 |  | *Juncellus serotinus* | Netlet | 9.23 | 0.55 | 0.37 | 10.50 | 0.71 | 0.44 | Seed | No |
| 126 |  | *Kyllinga brevifolia* | Netlet | 31.15 | 0.64 | 0.49 | 358.75 | 0.75 | 0.43 | Fruit | Glume |
| 127 |  | *Scirpus juncoides* | Netlet | 5.45 | 0.20 | 0.22 | 262.50 | 0.27 | 0.35 | Fruit | Glume |
| 128 |  | *Scirpus planiculmis* | Netlet | 27.16 | 0.80 | 0.47 | 7.24 | 0.83 | 0.27 | Fruit | Glume |
| 129 |  | *Scirpus wallichii* | Netlet | 35.38 | 0.37 | 0.82 | 328.72 | 0.75 | 0.49 | Fruit | Glume |
| 130 | Brassicaceae | *Capsella bursa-pastoris* | Silique | 20.40 | 0.90 | 0.27 | 13.33 | 0.83 | 0.38 | Seed | No |
| 131 |  | *Chorispora tenella* | Silique | 32.57 | 0.70 | 0.58 | 335.48 | 0.74 | 0.49 | Seed | No |
| 132 |  | *Descurainia sophia* | Silique | 30.00 | 0.74 | 0.64 | 331.36 | 0.79 | 0.44 | Seed | No |
| 133 |  | *Erysimum bungei* | Silique | 30.26 | 0.77 | 0.60 | 331.70 | 0.83 | 0.50 | Seed | No |
| 134 |  | *Lepidium apetalum* | Silique | 24.63 | 0.79 | 0.47 | 333.10 | 0.56 | 0.40 | Seed | No |
| 135 |  | *Rorippa cantoniensis* | Silique | 26.40 | 0.84 | 0.23 | 7.00 | 0.92 | 0.51 | Seed | No |
| 136 |  | *Rorippa globosa* | Silique | 32.31 | 0.55 | 0.65 | 331.00 | 0.94 | 0.50 | Seed | No |
| 137 |  | *Rorippa indica* | Silique | 30.64 | 0.80 | 0.36 | 12.96 | 0.86 | 0.57 | Seed | No |
| 139 | Polygonaceae | *Rumex trisetifer* | Achene | 33.00 | 0.75 | 0.53 | 358.25 | 0.59 | 0.68 | Fruit | No |
| 140 |  | *Polygonum lapathifolium* | Achene | 102.86 | 0.18 | 0.16 | 71.74 | 0.88 | 0.41 | Fruit | No |
| 141 |  | *Polygonum lapathifolium* var. *salicifolium* | Achene | 31.33 | 0.63 | 0.71 | 348.87 | 0.60 | 0.63 | Fruit | Tepals |
| 142 |  | *Polygonum orientale* | Achene | 30.00 | 0.16 | 0.29 | 29.43 | 0.55 | 0.38 | Fruit | No |
| 143 |  | *Polygonum perfoliatum* | Achene | 96.00 | 0.14 | 0.14 | 58.38 | 0.66 | 0.22 | Fruit | No |
| 144 |  | *Polygonum pubescens* | Achene | 75.00 | 0.13 | 0.24 | 63.21 | 0.90 | 0.49 | Fruit | No |
| 145 |  | *Rumex crispus* | Achene | 18.95 | 0.66 | 0.23 | 16.55 | 0.45 | 0.25 | Fruit | No |
| 146 |  | *Rumex dentatus* | Achene | 22.42 | 0.80 | 0.28 | 344.03 | 0.53 | 0.59 | Fruit | Tepals |
| 147 |  | *Rumex japonicus* | Achene | 23.40 | 0.69 | 0.44 | 9.92 | 0.78 | 0.61 | Fruit | No |
| 148 | Caryophyllaceae | *Cerastium viscosum* | Capsule | 35.73 | 0.46 | 0.76 | 341.57 | 0.86 | 0.64 | Seed | No |
| 149 |  | *Malachium aquaticum* | Capsule | 27.74 | 0.79 | 0.33 | 350.22 | 0.69 | 0.52 | Seed | No |
| 150 |  | *Silene conoidea* | Capsule | 39.13 | 0.63 | 0.35 | 356.35 | 0.70 | 0.47 | Seed | No |
| 151 |  | *Stellaria pusilla* | Capsule | 29.13 | 0.45 | 0.41 | 5.60 | 0.82 | 0.71 | Seed | No |
| 153 | Scrophulariaceae | *Lindernia crustacea* | Capsule | 32.73 | 0.63 | 0.41 | 344.64 | 0.84 | 0.58 | Seed | No |
| 154 |  | *Mazus japonicus* | Capsule | 43.19 | 0.73 | 0.32 | 345.53 | 0.85 | 0.39 | Seed | No |
| 155 |  | *Veronica polita* | Capsule | 60.00 | 0.20 | 0.20 | 283.64 | 0.48 | 0.18 | Seed | No |
| 160 | Convolvulaceae | *Calystegia hederacea* | Capsule | 218.57 | 0.26 | 0.21 | 20.45 | 0.69 | 0.25 | Seed | No |
| 161 |  | *Convolvulus arvensis* | Capsule | 26.67 | 0.11 | 0.31 | 343.11 | 0.72 | 0.56 | Seed | No |
| 166 | Apiaceae | *Cnidium monnier* | Cremocarp | 35.80 | 0.51 | 0.68 | 351.97 | 0.60 | 0.82 | Fruit | Winged ribs |
| 167 |  | *Sium suave* | Cremocarp | 36.15 | 0.66 | 0.45 | 351.37 | 0.68 | 0.67 | Fruit | Winged ribs |
| 168 |  | *Torilis scabra* | Cremocarp | 38.50 | 0.49 | 0.46 | 342.63 | 0.68 | 0.65 | Fruit | Aculeus |
| 171 | Amaranthaceae | *Achyranthes bidentata* | Utricle | 42.32 | 0.55 | 0.53 | 39.72 | 0.40 | 0.48 | Fruit | Bracteoles and tepals |
| 178 | Ranunculaceae | *Ranunculus ternatus* | Aggregated achenes | 58.70 | 0.74 | 0.49 | 346.71 | 0.80 | 0.68 | Fruit | Beak |
| 179 |  | *Ranunculus chinensis* | Aggregated achenes | 37.06 | 0.70 | 0.57 | 351.43 | 0.73 | 0.67 | Fruit | Beak |
| 180 |  | *Ranunculus sieboldii* | Aggregated achenes | 41.74 | 0.59 | 0.76 | 345.00 | 0.65 | 0.75 | Fruit | Beak |
| 181 | Rosaceae | *Potentilla supina* | Achene | 37.37 | 0.75 | 0.43 | 359.30 | 0.80 | 0.66 | Fruit | No |
| 184 | Solanaceae | *Solanum nigrum* | Berry | 33.40 | 0.53 | 0.78 | 325.71 | 0.75 | 0.36 | Seed | No |
| 186 | Commelinaceae | *Commelina communis* | Capsule | 67.50 | 0.05 | 0.58 | 332.43 | 0.84 | 0.52 | Seed | No |
| 187 |  | *Murdannia triquetra* | Capsule | 103.64 | 0.06 | 0.74 | 319.80 | 0.66 | 0.60 | Seed | No |
| 188 | Boraginaceae | *Bothriospermum chinense* | Nutlet | 28.18 | 0.46 | 0.56 | 338.11 | 0.95 | 0.31 | Fruit | Tuberculate protrusions |
| 189 |  | *Trigonotis peduncularis* | Nutlet | 25.00 | 0.59 | 0.48 | 324.12 | 0.78 | 0.51 | Fruit | Calyx |
| 190 | Alismataceae | *Sagittaria pygmaea* | Achene | 36.08 | 0.66 | 0.48 | 355.35 | 0.51 | 0.67 | Fruit | Wings |
| 191 |  | *Sagittaria sagittifolia* | Achene | 40.41 | 0.60 | 0.64 | 355.33 | 0.67 | 0.53 | Fruit | Wings |
| 192 |  | *Sagittaria trifolia* | Achene | 41.14 | 0.64 | 0.64 | 343.16 | 0.60 | 0.75 | Fruit | Wings |
| 193 |  | *Sagittaria trifolia* f. *longlioba* | Achene | 40.74 | 0.62 | 0.51 | 350.91 | 0.43 | 0.61 | Fruit | Wings |
| 194 | Geraniaceae | *Erodium stephanianum* | Capsule | 45.52 | 0.28 | 0.40 | 328.13 | 0.70 | 0.54 | Seed | No |
| 196 | Cannabaceae | *Cannabis sativa* | Achene | 35.00 | 0.68 | 0.62 | 348.92 | 0.85 | 0.72 | Fruit | Bracts |
| 197 |  | *Humulus scandens* | Achene | 33.91 | 0.57 | 0.43 | 356.56 | 0.69 | 0.89 | Fruit | No |
| 198 | Chenopodiaceae | *Chenopodium serotinum* | Utricle | 45.81 | 0.45 | 0.49 | 356.69 | 0.60 | 0.82 | Fruit | No |
| 199 |  | *Chenopodium album* | Utricle | 57.43 | 0.39 | 0.21 | 264.81 | 0.78 | 0.16 | Fruit | No |
| 200 |  | *Chenopodium glaucum* | Utricle | 27.13 | 0.49 | 0.19 | 353.56 | 0.64 | 0.20 | Fruit | No |
| 201 | Rubiaceae | *Galium aparine* | Achene | 32.18 | 0.68 | 0.32 | 350.97 | 0.60 | 0.57 | Fruit | Uncinated trichomes |
| 202 | Guttiferae | *Hypericum japonicum* | Capsule | 40.49 | 0.64 | 0.75 | 340.91 | 0.89 | 0.68 | Seed | No |
| 203 | Juncaceae | *Juncus effusus* | Caryopsis | 39.57 | 0.59 | 0.62 | 357.55 | 0.68 | 0.56 | Seed | No |
| 204 | Pontederiaceae | *Monochoria vaginalis* | Capsule | 40.37 | 0.46 | 0.40 | 352.45 | 0.92 | 0.44 | Seed | No |
| 205 | Plantaginaceae | *Plantago asiatica* | Capsule | 39.97 | 0.68 | 0.19 | 62.13 | 0.39 | 0.17 | Seed | No |
| 206 | Potamogetonaceae | *Potamogeton distinctus* | Drupe | 37.69 | 0.55 | 0.56 | 331.96 | 0.63 | 0.67 | Fruit | Keels and beak |
| 208 | Balsaminaceae | *Impatiens balsamina* | Capsule | 30.81 | 0.53 | 0.27 | 341.14 | 0.85 | 0.49 | Seed | No |

Table 2 HSV color space of pre-stained and stained seeds/fruits of invasion plants

| Serial number | Family | Latin Name | Seed type | Unstained | | | Stained | | | Seed/Fruit | Appendage |
| --- | --- | --- | --- | --- | --- | --- | --- | --- | --- | --- | --- |
|  |  |  |  | H1 | S1 | V1 | H2 | S2 | V2 |  |  |
| 44 | Poaceae | *Alopecurus japonicus* | Caryopsis | 63.46 | 0.40 | 0.65 | 351.08 | 0.70 | 0.82 | Fruit | Lemma |
| 45 |  | *Arthraxon hispidus* | Caryopsis | 42.60 | 0.47 | 0.63 | 344.70 | 0.74 | 0.79 | Fruit | Lemma |
| 46 |  | *Bromus catharticus* | Caryopsis | 41.88 | 0.52 | 0.73 | 327.43 | 0.85 | 0.64 | Fruit | Lemma and palea |
| 47 |  | *Echinochloa crusgalli* | Caryopsis | 45.00 | 0.53 | 0.62 | 350.73 | 0.77 | 0.56 | Fruit | Lemma |
| 48 |  | *Eleusine indica* | Caryopsis | 53.33 | 0.20 | 0.52 | 328.29 | 0.81 | 0.60 | Fruit | Lemma and palea |
| 49 |  | *Imperata cylindrica* | Caryopsis | 32.31 | 0.48 | 0.53 | 339.07 | 0.74 | 0.68 | Fruit | Lemma and palea |
| 50 |  | *Lolium multiflorum* | Caryopsis | 36.80 | 0.51 | 0.58 | 336.60 | 0.74 | 0.75 | Fruit | Lemma and palea |
| 51 |  | *Lolium perenne* | Caryopsis | 40.22 | 0.45 | 0.80 | 329.72 | 0.84 | 0.51 | Fruit | Lemma and palea |
| 52 |  | *Lolium temulentum* | Caryopsis | 41.88 | 0.46 | 0.82 | 324.66 | 0.89 | 0.72 | Fruit | Lemma and palea |
| 53 |  | *Lolium temulentum* var. *arvense* | Caryopsis | 37.21 | 0.70 | 0.72 | 334.34 | 0.85 | 0.70 | Fruit | Lemma and palea |
| 54 |  | *Lolium temulentum* var. *longiaristatum* | Caryopsis | 46.67 | 0.60 | 0.47 | 347.52 | 0.78 | 0.75 | Fruit | Lemma and palea |
| 55 |  | *Paspalum dilatatum* | Caryopsis | 43.27 | 0.69 | 0.84 | 333.28 | 0.82 | 0.62 | Fruit | Lemma |
| 56 |  | *Sorghum halepense* | Caryopsis | 3.16 | 0.46 | 0.16 | 306.21 | 0.70 | 0.33 | Fruit | Lemma |
| 57 |  | *Sorghum sudanense* | Caryopsis | 15.00 | 0.22 | 0.14 | 302.07 | 0.59 | 0.19 | Fruit | Lemma |
| 70 | Asteraceae | Ageratina adenophora | Achene | 64.29 | 0.22 | 0.25 | 357.72 | 0.90 | 0.69 | Fruit | Pappi |
| 71 |  | *Ageratum conyzoides* | Achene | 43.00 | 0.43 | 0.54 | 335.38 | 0.69 | 0.44 | Fruit | Pappi |
| 72 |  | *Ambrosia artemisiifolia* | Achene | 38.24 | 0.56 | 0.64 | 343.45 | 0.49 | 0.46 | Fruit | Pappi |
| 73 |  | *Ambrosia trifida* | Achene | 25.86 | 0.70 | 0.33 | 22.89 | 0.97 | 0.31 | Fruit | Involucre |
| 74 |  | *Artemisia capillaries* | Achene | 36.73 | 0.74 | 0.52 | 343.55 | 0.79 | 0.62 | Fruit | Pappi |
| 75 |  | *Aster subulatus* | Achene | 36.79 | 0.62 | 0.67 | 345.80 | 0.89 | 0.58 | Fruit | Pappi |
| 76 |  | *Bidens pilosa* | Achene | 35.63 | 0.46 | 0.27 | 16.36 | 0.62 | 0.28 | Fruit | Barbed awns |
| 77 |  | *Centaurea cyanus* | Achene | 32.61 | 0.86 | 0.63 | 353.47 | 0.72 | 0.55 | Fruit | Pappi |
| 78 |  | *Cichorium intybus* | Achene | 32.57 | 0.58 | 0.47 | 12.73 | 0.28 | 0.35 | Fruit | Pappi |
| 79 |  | *Conyza canadensis* | Achene | 38.06 | 0.65 | 0.56 | 345.74 | 0.85 | 0.56 | Fruit | Pappi |
| 80 |  | *Coreopsis lanceolata* | Achene | 32.83 | 0.53 | 0.39 | 343.61 | 0.92 | 0.78 | Fruit | Wings |
| 81 |  | *Eupatorium odoratum* | Achene | 38.36 | 0.54 | 0.45 | 340.60 | 0.90 | 0.58 | Fruit | Pappi |
| 82 |  | *Flaveria bidentis* | Achene | 63.00 | 0.24 | 0.33 | 9.47 | 0.74 | 0.30 | Fruit | No |
| 83 |  | *Silybum marianum* | Achene | 70.00 | 0.14 | 0.84 | 329.01 | 0.78 | 0.76 | Fruit | Pappi |
| 84 |  | *Solidago canadensis* | Achene | 30.57 | 0.34 | 0.77 | 344.54 | 0.60 | 0.66 | Fruit | Pappi |
| 85 |  | *Sonchus asper* | Achene | 30.00 | 0.73 | 0.45 | 336.18 | 0.41 | 0.65 | Fruit | Pappi |
| 86 |  | *Synedrella nodiflora* | Achene | 43.37 | 0.58 | 0.69 | 341.67 | 0.54 | 0.53 | Fruit | Pappi |
| 99 | Euphorbiaceae | *Euphorbia cyathophora* | Capsule | 40.68 | 0.67 | 0.69 | 341.36 | 0.75 | 0.69 | Fruit | No |
| 100 |  | *Euphorbia helioscopia* | Capsule | 8.78 | 0.48 | 0.33 | 330.77 | 0.59 | 0.26 | Seed | No |
| 101 |  | *Euphorbia hirta* | Capsule | 33.66 | 0.49 | 0.65 | 347.50 | 0.91 | 0.72 | Seed | No |
| 102 |  | *Euphorbia maculata* | Capsule | 38.90 | 0.57 | 0.35 | 341.47 | 0.89 | 0.65 | Fruit | No |
| 103 |  | *Euphorbia nutans* | Capsule | 39.46 | 0.51 | 0.33 | 351.47 | 0.79 | 0.55 | Fruit | No |
| 112 | Leguminosae | *Cassia tora* | Pod | 13.85 | 0.37 | 0.27 | 19.41 | 0.64 | 0.42 | Seed | No |
| 113 |  | *Lupinus luteus* | Pod | 47.69 | 0.36 | 0.85 | 341.40 | 0.49 | 0.81 | Seed | No |
| 114 |  | *Medicago minima* | Pod | 40.00 | 0.62 | 0.55 | 352.45 | 0.77 | 0.77 | Fruit | Hooked spines |
| 115 |  | *Medicago polymorpha* | Pod | 36.89 | 0.46 | 0.27 | 10.82 | 0.59 | 0.41 | Fruit | Spines or tubercles |
| 116 |  | *Medicago sativa* | Pod | 17.56 | 0.67 | 0.48 | 1.17 | 0.78 | 0.52 | Seed | No |
| 117 |  | *Mimosa pudica* | Pod | 24.80 | 0.49 | 0.60 | 350.94 | 0.79 | 0.69 | Fruit | No |
| 156 | Scrophulariaceae | *Veronica peregrina* | Capsule | 26.67 | 0.52 | 0.34 | 316.07 | 0.57 | 0.38 | Seed | No |
| 157 |  | *Veronica hederaefolia* | Capsule | 38.82 | 0.30 | 0.22 | 282.86 | 0.47 | 0.18 | Seed | No |
| 158 |  | *Veronica arvensis* | Capsule | 31.84 | 0.88 | 0.42 | 3.97 | 0.79 | 0.60 | Seed | No |
| 159 |  | *Veronica persica* | Capsule | 42.58 | 0.37 | 0.63 | 346.14 | 0.61 | 0.57 | Seed | No |
| 162 | Convolvulaceae | *Cuscuta campestris* | Capsule | 29.57 | 0.35 | 0.76 | 352.22 | 0.87 | 0.73 | Seed | No |
| 163 |  | *Ipomoea triloba* | Capsule | 15.00 | 0.24 | 0.20 | 349.57 | 0.30 | 0.30 | Seed | No |
| 164 |  | *Pharbitis nil* | Capsule | 210.97 | 0.37 | 0.33 | 6.52 | 0.37 | 0.49 | Seed | No |
| 165 |  | *Pharbitis purpurea* | Capsule | 220.00 | 0.38 | 0.15 | 16.80 | 0.37 | 0.27 | Seed | No |
| 169 | Apiaceae | *Chaerophyllum villosum* | Cremocarp | 26.84 | 0.68 | 0.66 | 354.59 | 0.79 | 0.60 | Fruit | No |
| 170 |  | *Daucus carota* | Cremocarp | 25.00 | 0.69 | 0.61 | 329.17 | 0.78 | 0.55 | Fruit | Bristles |
| 172 | Amaranthaceae | *Alternanthera philoxeroides* | Utricle | 35.70 | 0.66 | 0.71 | 343.76 | 0.83 | 0.63 | Fruit | No |
| 173 |  | *Amaranthus blitoides* | Utricle | 52.36 | 0.27 | 0.80 | 313.85 | 0.61 | 0.83 | Fruit | Perianth |
| 174 |  | *Amaranthus retroflexus* | Utricle | 42.20 | 0.48 | 0.74 | 346.44 | 0.87 | 0.65 | Fruit | Tepals |
| 175 |  | *Amaranthus spinosus* | Utricle | 353.33 | 0.32 | 0.11 | 30.00 | 0.39 | 0.18 | Seed | No |
| 176 |  | *Amaranthus viridis* | Utricle | 40.17 | 0.62 | 0.76 | 346.72 | 0.90 | 0.57 | Fruit | Tepals |
| 177 |  | *Celosia argentea* | Utricle | 240.00 | 0.04 | 0.09 | 48.00 | 0.56 | 0.07 | Seed | No |
| 195 | Geraniaceae | *Geranium carolinianum* | Capsule | 42.74 | 0.56 | 0.51 | 352.17 | 0.50 | 0.54 | Fruit | Glandular trichomes |
| 207 | Zygophyllaceae | *Tribulus terrester* | Capsule | 45.76 | 0.43 | 0.54 | 336.00 | 0.91 | 0.41 | Fruit | Thorns |
| 138 | Brassicaceae | *Brassica juncea* | Silique | 18.46 | 0.57 | 0.36 | 316.55 | 0.41 | 0.28 | Seed | No |
| 152 | Caryophyllaceae | *Vaccaria segetalis* | Capsule | 207.80 | 0.33 | 0.49 | 345.31 | 0.69 | 0.28 | Seed | No |
| 185 | Solanaceae | *Physalis angulata* | Berry | 21.55 | 0.57 | 0.71 | 354.96 | 0.77 | 0.60 | Seed | No |

Table 3 HSV color space of pre-stained and stained seeds/fruits of woody plants

| Serial number | Family | Latin Name | Fruit type | Unstained | | | Stained | | | Seed/Fruit | Appendage |
| --- | --- | --- | --- | --- | --- | --- | --- | --- | --- | --- | --- |
|  |  |  |  | H1 | S1 | V1 | H2 | S2 | V2 |  |  |
| 104 | Euphorbiaceae | *Glochidion puberum* | Capsule | 7.94 | 0.60 | 0.45 | 356.19 | 0.81 | 0.61 | Seed | No |
| 105 |  | *Fluggea suffruticosa* | Capsule | 20.00 | 0.94 | 0.14 | 357.30 | 0.91 | 0.38 | Seed | No |
| 106 |  | *Phyllanthus urinaria* | Capsule | 32.73 | 0.64 | 0.40 | 348.00 | 0.69 | 0.54 | Fruit | Sepals |
| 107 |  | *Sapium sebiferum* | Capsule | 50.40 | 0.32 | 0.62 | 349.02 | 0.56 | 0.58 | Seed | No |
| 108 |  | *Vernicia fordii* | Drupe | 39.05 | 0.56 | 0.44 | 353.48 | 0.68 | 0.53 | Seed | No |
| 118 | Leguminosae | *Caesalpinia decapetala* | Pod | 4.62 | 0.18 | 0.28 | 24.83 | 0.42 | 0.27 | Seed | No |
| 119 |  | *Cassia occidentalis* | Pod | 40.00 | 0.38 | 0.28 | 355.14 | 0.62 | 0.47 | Seed | No |
| 120 |  | *Leucaena leucocephala* | Pod | 353.79 | 0.34 | 0.34 | 0.94 | 0.50 | 0.50 | Seed | No |
| 209 | Magnoliaceae | *Liriodendron chinense* | Nutlet | 40.99 | 0.56 | 0.71 | 338.00 | 0.70 | 0.67 | Fruit | Beak |
| 210 |  | *Michelia alba* | Follicle | 6.85 | 0.82 | 0.71 | 354.00 | 0.62 | 0.38 | Seed | No |
| 211 | Fagaceae | *Cyclobalanopsis glauca* | Nut | 32.18 | 0.63 | 0.68 | 333.95 | 0.64 | 0.79 | Fruit | Bract |
| 212 |  | *Quercus acutissima* | Nut | 32.09 | 0.40 | 0.42 | 348.71 | 0.64 | 0.62 | Fruit | Bract |
| 182 | Rosaceae | *Rosa multiflora* | Achene | 277.50 | 0.22 | 0.14 | 273.75 | 0.46 | 0.27 | Fruit | No |
| 183 |  | *Rosa roxburghii* | Achene | 25.65 | 0.81 | 0.63 | 344.32 | 0.74 | 0.58 | Fruit | Sepals |
| 213 | Juglandaceae | *Carya illinoinensis* | Nut | 34.82 | 0.64 | 0.68 | 355.71 | 0.54 | 0.51 | Fruit | No |
| 214 |  | *Pterocarya stenoptera* | Samara | 37.36 | 0.49 | 0.43 | 340.00 | 0.63 | 0.52 | Fruit | Wings |
| 215 | Aceraceae | *Acer buergerianum* | Samara | 30.76 | 0.36 | 0.66 | 337.30 | 0.70 | 0.65 | Fruit | Wings |
| 216 |  | *Acer fabri* | Samara | 52.50 | 0.17 | 0.56 | 355.26 | 0.47 | 0.64 | Fruit | Wings |
| 217 |  | *Acer palmatum* | Samara | 48.73 | 0.29 | 0.78 | 327.79 | 0.59 | 0.65 | Fruit | Wings |
| 218 | Hippocastanaceae | *Aesculus turbinata* | Capsule | 37.30 | 0.76 | 0.57 | 347.14 | 0.67 | 0.57 | Fruit | No |
| 219 | Simaroubaceae | *Ailanthus altissima* | Samara | 44.57 | 0.61 | 0.67 | 358.24 | 0.55 | 0.49 | Fruit | Wings |
| 220 | Verbenaceae | *Callicarpa bodinieri* | Berry | 13.40 | 0.86 | 0.43 | 351.74 | 0.86 | 0.50 | Fruit | No |
| 221 | Nyssaceae | *Camptotheca acuminata* | Samara | 36.27 | 0.63 | 0.83 | 358.95 | 0.60 | 0.74 | Fruit | Wings |
| 222 | Betulaceae | *Corylus heterophylla* | Nut | 28.97 | 0.74 | 0.46 | 350.64 | 0.69 | 0.62 | Fruit | Bracts |
| 223 | Saxifragaceae | *Deutzia scabra* | Capsule | 61.09 | 0.31 | 0.69 | 335.06 | 0.68 | 0.51 | Fruit | No |
| 224 | Ebenaceae | *Diospyros rhombifolia* | Berry | 31.13 | 0.86 | 0.96 | 348.26 | 0.69 | 0.52 | Fruit | No |
| 225 | Celastraceae | *Euonymus fortunei* | Capsule | 17.47 | 0.97 | 0.74 | 344.37 | 0.73 | 0.64 | Fruit | No |
| 226 | Berberidaceae | *Nandina domestica* | Berry | 54.41 | 0.83 | 0.56 | 343.11 | 0.72 | 0.56 | Fruit | No |
| 227 | Lauraceae | *Phoebe sheareri* | Berry | 55.71 | 0.27 | 0.20 | 12.00 | 0.14 | 0.14 | Seed | No |
| 228 | Cupressaceae | *Platycladus orientalis* | Cone | 26.58 | 0.76 | 0.41 | 11.49 | 0.50 | 0.37 | Seed | No |
| 229 | Palmae | *Trachycarpus fortunei* | Drupe | 57.86 | 0.31 | 0.35 | 334.74 | 0.43 | 0.17 | Seed | No |
| 230 | Ulmaceae | *Ulmus parvifolia* | Samara | 24.92 | 0.63 | 0.41 | 335.24 | 0.62 | 0.40 | Fruit | Wings |
| 231 | Apocynaceae | *Apocynum venetum* | Capsule | 31.58 | 0.52 | 0.29 | 353.25 | 0.63 | 0.50 | Fruit | Pappi |
| 232 | Eucommiaceae | *Eucommia ulmoides* | Nut | 49.87 | 0.42 | 0.69 | 355.92 | 0.69 | 0.70 | Fruit | Wings |
| 233 | Lythraceae | *Lagerstroemia indica* | Cone | 38.89 | 0.38 | 0.55 | 341.14 | 0.63 | 0.44 | Fruit | Wings |
