# Supplementary material for: A new technique for stain-marking of seeds with safranine to track seed dispersal and seed bank dynamics
Source: Front Plant Sci. 2022 Aug 8;13:959046. doi: 10.3389/fpls.2022.959046 (PMC9393530; doi:10.3389/fpls.2022.959046)
Supplement: Supplementary file 3 [file Data_Sheet_3.docx]

Table 4 Code example for diaspores deification and counting

| import cv2  import numpy as np  imagepath1= "C:\\Users\\jay\\Desktop\\Photos\\Mixed\\1.png"  img1 = cv2.imread(imagepath1, cv2.IMREAD_UNCHANGED)  # (1) Color space conversion (GBR to HSV)  imghsv = cv2.cvtColor(img1,cv2.COLOR_BGR2HSV)  # (2)Definition of target color of stained diaspores  Lower1 = np.array([125,43,46])  Lower2 = np.array([0,43,46])  Upper1 = np.array([180,255,255])  Upper2 = np.array([10,255,255])  # (3) Mask obtaining of binary image  mask_1 = cv2.inRange(imghsv, Lower1, Upper1)  mask_2 = cv2.inRange(imghsv, Lower2, Upper2)  mask0= cv2.add(mask_1,mask_2)  res = cv2.bitwise_and(img1, img1, mask=mask0)  blurred = cv2.blur(res,(10,10))  blurred = cv2.cvtColor(blurred,cv2.COLOR_BGR2HSV)  mask1 = cv2.inRange(blurred, Lower1, Upper1)  mask2 = cv2.inRange(blurred, Lower2, Upper2)  mask= cv2.add(mask1,mask2)  # (4) Morphological processing  kernel = cv2.getStructuringElement(cv2.MORPH_RECT, (20, 20))  Opened=cv2.morphologyEx(mask, cv2.MORPH_OPEN, kernel)  kernel2 = cv2.getStructuringElement(cv2.MORPH_RECT, (5, 5))  Closed2=cv2.morphologyEx(Opened,cv2.MORPH_CLOSE, kernel2)  # (5) Contours discovery and numbering of found contours on photos  binary,contours,hierarchy = cv2.findContours(Closed2,cv2.RETR_EXTERNAL, cv2.CHAIN_APPROX_SIMPLE)  print("number of diaspores: %d" % (len(contours)))  img1_copy= img1.copy()  cv2.drawContours(img1_copy, contours,-1, (0,255,0),1)  for i in range(len(contours)):  rect = cv2.minAreaRect(contours[i])  x,y = rect[0]  center = (int(x-20), int(y+10))  angle = rect[2]  font=cv2.FONT_HERSHEY_SIMPLEX  cv2.putText(img1_copy,str(i+1),center,font,1,(0,255,0),1)  cv2.imshow('Result1', img1_copy)  cv2.waitKey(0)  cv2.imwrite('Result1.jpg',img1_copy)  cv2.putText(img1_copy,("number of contours: %d" % (len(contours))),(200,200),font,2,(0,255,0),2) |
| --- |


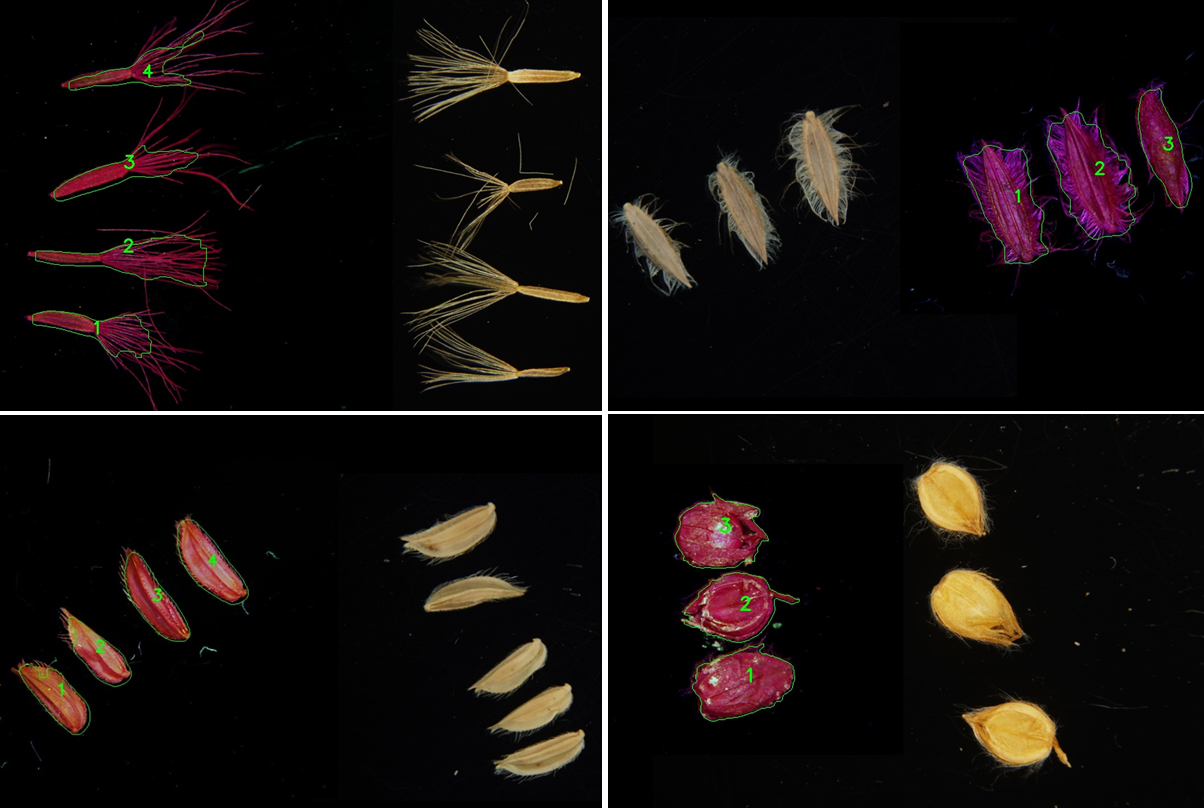


Figure 9 Examples of the indemnification of stained diaspores using openCV on python platform
